# High accuracy inverse design of reconfigurable metasurfaces with transmission-reflection-integrated achromatic functionalities

**DOI:** 10.1515/nanoph-2024-0680

**Published:** 2025-03-25

**Authors:** Xiao-Qiang Jiang, Wen-Hui Fan, Xu Chen, Lv-Rong Zhao, Chong Qin, Hui Yan, Qi Wu, Pei Ju

**Affiliations:** State Key Laboratory of Transient Optics and Photonics, Xi’an Institute of Optics and Precision Mechanics, Chinese Academy of Sciences, Xi’an 710119, P.R. China; University of Chinese Academy of Sciences, Beijing 100049, P.R. China; Collaborative Innovation Center of Extreme Optics, Shanxi University, Taiyuan 030006, P.R. China

**Keywords:** terahertz, metasurfaces, deep learning, vortex beam, imaging

## Abstract

Artificial intelligence algorithms based on deep neural network (DNN) have become an effective tool for conceiving metasurfaces recently. However, the complex and sharp resonances of metasurfaces will tremendously increase the training difficulty of DNNs with non-negligible prediction errors, which hinders their development in designing multifunctional metasurfaces. To overcome the obstacles, the interaction mechanisms between meta-atoms and terahertz (THz) waves via multipole decomposition are investigated to establish a high-quality dataset, which can decrease the complexity of DNN and improve the prediction accuracy. Meanwhile, transfer learning is also employed to reduce the large quantity of training data required by the DNN. Accordingly, two broadband and transmission-reflection-integrated reconfigurable metasurfaces for focused vortex beam generation are inversely designed by counter propagating the DNN with fraction error less than 10^−4^. The results indicate that transmission-reflection-integrated achromatic performances are well achieved in the frequency range of 0.7–1.3 THz, which have the average focusing efficiency and mode purity higher than 48 % and 92 %, respectively. Moreover, transmission-reflection-integrated achromatic THz imaging and edge detection can also be realized by the metasurfaces. This work provides a high accuracy inverse design method for conceiving multifunctional meta-devices, which may promise further progress for the on-chip THz imaging systems.

## 1 Introduction

Terahertz (THz) waves, with frequency range of 0.1–10 THz in the electromagnetic (EM) spectrum, have demonstrated numerous promising applications that can greatly reshape future researches and industrial fields [1], [2], [3]. As for the further exploitation of THz waves, it can be realized by manipulating their wavefronts to obtain desired special beams [4], [5]. Therefore, THz waves carrying additional EM characteristics can greatly extend their application boundaries. For instance, THz vortex beams carrying orbital angular momentum (OAM) make them extraordinarily attractive in terabit wireless communications due to the mutually orthogonal eigenstates of OAM [6], [7]. Besides, the helical phase-fronts and hollow properties of THz vortex beams can realize super-resolution imaging and edge detection [8], [9]. Specifically, the spiral phase contrast (SPC) imaging via OAM is of great importance in edge detection since it can reconstruct the edge information for both amplitude and phase objects, which may have great potential in machine vision and remote sensing [10], [11]. Until now, plentiful methods have been implemented for generating optical vortex beams, including spiral phase plates and spatial light modulators [7], [12]. However, these conventional devices confront great challenges in THz range since natural materials barely have EM responses to THz waves, which severely impedes the further development of THz technology.

Due to the powerful capabilities for manipulating EM waves, metasurfaces may have considerable potential to realize revolutionary advancements that overcome the constraints of natural materials on the development of THz technology [13], [14], [15]. By employing metasurfaces, the generation of THz vortex beams and their applications have been developed rapidly [5], [16]. However, these devices still confront limited function, which can only be operated in either transmission mode (T-mode) or reflection mode (R-mode), greatly wasting space resources [17]. To realize broadband achromatic focusing in both T-mode and R-mode, multi-layer metallic metasurfaces with frequency- and polarization-multiplexing capabilities were proposed [18], [19]. Nevertheless, the focusing efficiencies of these transmission-reflection-integrated metasurfaces are only about 20 % due to the complex structures and strict operating requirements. Although monolayer dielectric metasurfaces were conducted for transmission-reflection-integrated optical imaging, they still confront inefficiency and limited bandwidth in the implementations [20], [21]. In contrast with passive counterparts, reconfigurable metasurfaces with dynamic properties can enable more flourishing performances, becoming a major frontier in further development of metasurfaces [22], [23]. Since vanadium dioxide (VO_2_) can span five orders of magnitude changes in its conductivity during the insulator-to-metal transition (IMT) [24], the transmission-reflection-integrated operation can be accomplished by employing VO_2_ instead of complicated multi-layer structures.

In order to accomplish transmission-reflection-integrated achromatic metasurfaces, each meta-atom is expected to satisfy the phase requirements in both T-mode and R-mode with relatively high efficiency. The complicated demands and in-depth connections are difficult to be recognized by limited human brainpower, which becomes a common issue in engineering sophisticated meta-devices [25]. Recently, deep learning has been employed to solve the bottlenecks [26], [27]. However, current deep neural networks (DNNs) are primarily focused on amplitude-modulation metasurfaces due to their relatively simple EM responses [28], [29], [30]. Since both amplitude and phase responses should be considered in the design of phase-modulation metasurfaces, their training difficulty is much harder than that of amplitude-modulation metasurfaces [31]. Besides, the complicated resonances will also cause non-negligible prediction errors. For instance, previous research for phase prediction suffered from test error up to 16° due to the sharp peaks [32]. This defect is still one of the most severe obstacles to design phase-modulation metasurfaces by DNNs [33], [34]. Although several methods were proposed by topological optimization or other advanced algorithms, their performances are highly depended on algorithm itself and require a large amount of training data and testing time. More importantly, the EM responses of meta-atoms barely receive enough attention, which are the foundation for designing metasurfaces. In general, the inverse design of broadband metasurfaces with advanced functionalities is still a huge challenge due to the complex spectral responses and phase requirements.

In this work, a high accuracy inverse design method is proposed to accomplish transmission-reflection-integrated achromatic reconfigurable metasurface (TRARM) for the first time to our knowledge. To satisfy complicated phase requirements, DNN and transfer learning are employed in the design scheme. Moreover, the fundamental mechanisms of interactions between meta-atoms and THz waves are investigated via multipole decomposition to exclude undesirable resonances. Thus, a high-quality dataset can be established to decrease the complexity of DNN and improve its prediction accuracy. To validate the inverse design method, two focused vortex beam (FVB) generators with different parameters are constructed and marked as TRARM-Ⅰ and TRARM-Ⅱ, respectively. Numerical results indicate that chromatic aberrations in both R-mode (0.7–1.0 THz) and T-mode (1.0–1.3 THz) can be well corrected. The maximum deviation ratios of TRARM-Ⅰ are 5.14 % (R-mode) and 4.30 % (T-mode), while that of TRARM-Ⅱ are 7.73 % (R-mode) and 4.32 % (T-mode). The average focusing efficiencies of these two metasurfaces are all higher than 48 %, and the OAM mode purity of TRARM-Ⅱ is higher than 92 %. Moreover, transmission-reflection-integrated achromatic THz imaging and edge detection can be also achieved by the metasurfaces. The presented high accuracy inverse design method has great significance in conceiving multifunctional metasurfaces.

## 2 Design principle for TRARM

**Figure 1:**
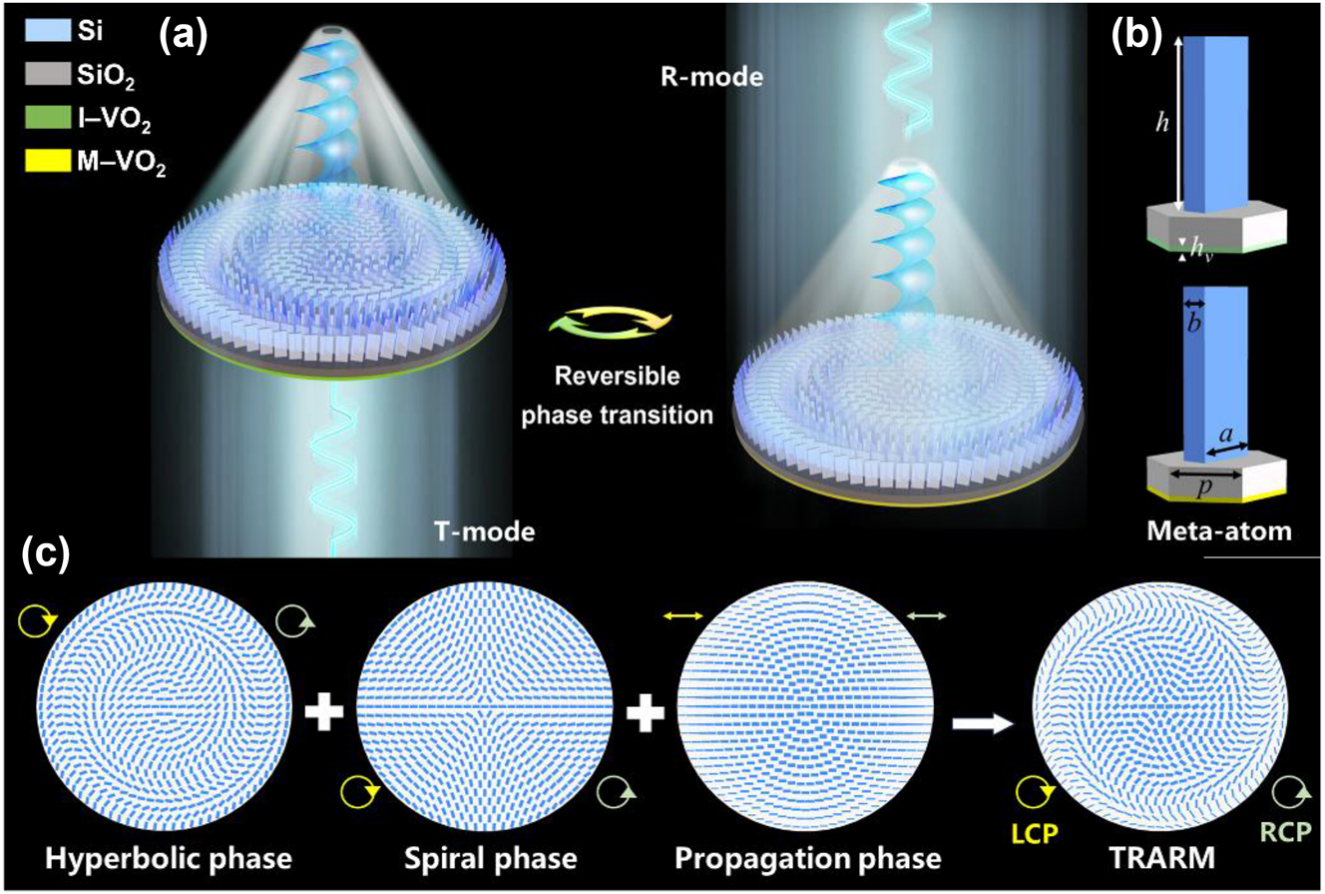
Schematics of (a) TRARM, (b) meta-atoms and (c) phase profiles. The yellow and green circles represent the polarization states of incident and outgoing THz waves, respectively.

The operation schematics of TRARM are illustrated in Figure 1(a), which can generate FVB and correct chromatic aberration in both T-mode (insulator phase, I-VO_2_) and R-mode (metallic phase, M-VO_2_), and it is a reversible transition. The polarization state of incident THz waves are left-circularly polarization (LCP), and that of generated FVBs are right-circularly polarization (RCP). The meta-atoms with lattice constant *p*, VO_2_ thickness *h*_*v*_, pillar height *h*, length *a*, and width *b* are shown in Figure 1(b), which consists of a high-resistance silicon (Si) block on silicon dioxide (SiO_2_) hexagonal substrate and VO_2_ is introduced as a bottom plate. The design principle of TRARM is shown in Figure 1(c), composed of three phase profiles of hyperbolic phase, spiral phase, and propagation phase, which are responsible for converging THz waves, generating vortex beam, and correcting chromatic aberration, respectively.

Benefitting from extraordinary capabilities of metasurfaces in manipulating EM waves, the hyperbolic phase and spiral phase can be super-imposed on a monolayer metasurface [35]:

(1) $$\varphi_{b}\left(x,y,f\right)=\frac{2\pi f}{c}\left(\sqrt{x^{2}+y^{2}+{F_{l}}^{2}}-F_{l}\right)+l\cdot \arctan\left(\frac{y}{x}\right)$$

where *f* represents the operating frequency, *c* denotes the light speed in vacuum, *l* is the topological charge of OAM, and *F*_*l*_ is the focal length. To correct chromatic aberration in the broadband frequency range, an additional phase compensation should be introduced [36]. Nevertheless, current broadband design method is employed to compensate the phase difference between *f* and *f*_min_ (minimum frequency) [37], which is not applicable to design the transmission-reflection-integrated achromatic metasurfaces. Therefore, the design method should be further improved for designing TRARM, and the modified phase profiles are expressed as:

(2) $$\varphi_{r}\left(x,y,f_{r}\right)=\varphi_{br}\left(x,y,f_{\text{maxr}}\right)+\Delta \varphi_{r}\left(x,y,f_{r}\right)$$

(3) $$\varphi_{t}\left(x,y,f_{t}\right)=\varphi_{bt}\left(x,y,f_{\text{mint}}\right)+\Delta \varphi_{t}\left(x,y,f_{t}\right)$$

where the footnote max and min are maximum and minimum frequency, and the footnote *t* and *r* represent T-mode and R-mode of TRARM. And the frequency range of *f*_*r*_ and *f*_*t*_ are *f*_*r*_ ∈ {*f*_minr_, *f*_maxr_} and *f*_*t*_ ∈ {*f*_mint_, *f*_maxt_}, respectively. Therefore, the phase compensation Δ*φ* can be described as:

(4) $$\Delta \varphi_{r}\left(x,y,f_{r}\right)=\left[\frac{2\pi }{c}\left(\sqrt{x^{2}+y^{2}+F_{lr}^{2}}-F_{lr}\right)\right]\cdot \left(f_{\text{maxr}}-f_{r}\right)$$

(5) $$\Delta \varphi_{t}\left(x,y,f_{t}\right)=\left[\frac{2\pi }{c}\left(\sqrt{x^{2}+y^{2}+{F_{lt}}^{2}}-F_{lt}\right)\right]\cdot \left(f_{t}-f_{\text{mint}}\right)$$

**Figure 2:**
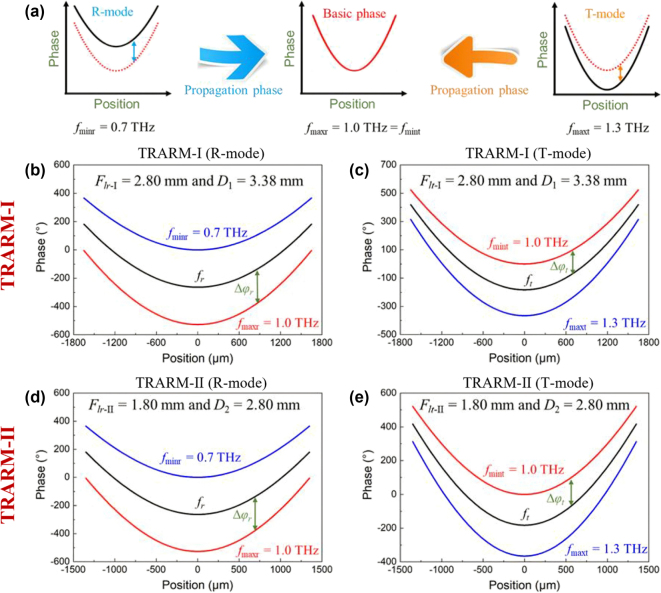
Phase requirements. (a) Illustration of phase compensation. Phase compensations for TRARM-Ⅰ in (b) R-mode and (c) T-mode. Phase compensations for TRARM-Ⅱ in (d) R-mode and (e) T-mode.

In Eqs. (2) and (3), *φ*_b_ is considered as basic phase for THz FVB generation and it can be satisfied by Pancharatnam–Berry (PB) phase for both modes. Meanwhile, Δ*φ* is responsible for correcting chromatic aberration by exploiting propagation phase. The illustration of phase compensation for T-mode and R-mode is depicted in Figure 2(a), where *f*_maxr_ and *f*_mint_ are both set as 1 THz with identical focal length. And the propagation phase is employed to compensate the phase differences between *f*_*r*_ (*f*_*t*_) and *f*_maxr_ (*f*_mint_) for R-mode (T-mode), respectively.

In this work, the focal length and diameter of TRARM-Ⅰ are set as *F*_*lt*-Ⅰ_ = *F*_*lr*-Ⅰ_ = 2.80 mm and *D*_1_ = 3.38 mm, and that of TRARM-Ⅱ are *F*_*lt*-Ⅱ_ = *F*_*lr-*Ⅱ_ = 1.80 mm and *D*_2_ = 2.80 mm. Meanwhile, the operating frequency ranges are 0.7–1.0 THz for R-mode and 1.0–1.3 THz for T-mode, respectively. The selected parameters and frequency range are explained in Supplementary Section 1. Accordingly, the phase compensations for TRARM-Ⅰ and TRARM-Ⅱ are depicted in Figure 2(b)–(e). Since *f*_maxr_ and *f*_mint_ are both set as 1 THz with identical focal length, the meta-atoms can share the same rotation angles at each position. Meanwhile, the phase compensation for TRARM in T-mode and R-mode should also be satisfied simultaneously by the meta-atoms, which is one of the major difficulties for designing TRARM. Fortunately, this obstruction can be readily solved by employing the proposed inverse design method.

## 3 High accuracy inverse design method

To resolve the defects of current DNNs in designing multifunctional phase-modulation metasurfaces, a high accuracy inverse design method via physical analyses, deep learning, and transfer learning is presented. To begin with, the multipole decomposition is performed to investigate the fundamental mechanisms of interactions between meta-atoms and THz waves, which can provide a high-quality dataset to significantly improve the prediction accuracy. Subsequently, DNN and transfer learning are both employed to predict the EM responses of meta-atoms and inversely design the TRARM.

### 3.1 Investigation on resonance mechanism

According to Eqs. (2)–(5), the phase responses of meta-atoms should be linearly proportional to operating frequency, indicating that resonances are not allowed to appear in the EM spectra. Moreover, the basic phase profile for R-mode is set at maximum frequency of 1.0 THz, requiring a large range of phase compensation to cover the full 2*π* phase range at minimum frequency of 0.7 THz. Therefore, the *p* and *h* of meta-atoms should be as large as possible to provide sufficient phase compensation. However, meta-atoms with large *p* and *h* can also induce undesired resonances to deteriorate their performances [38]. Therefore, the underlying physical mechanisms of EM responses from meta-atoms should be investigated to delicately design the *p* and *h*, which are expected to satisfy the phase requirements and avoid undesired resonances in the operating frequency range.

**Figure 3:**
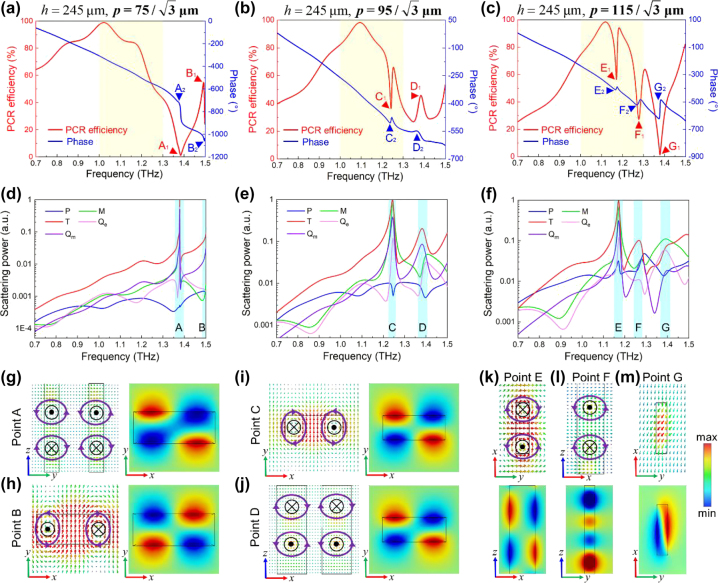
Optical responses of meta-atoms. (a)–(c) PCR efficiency and phase responses of meta-atoms. (d)–(f) Scattering power of multipole decomposition. (g)–(m) Magnetic field distributions. Black boxes are the outline of Si blocks, purple circles are the rotation of magnetic fields, and the black dots and forks are the outward and inward plane.

Since identical meta-atoms will have larger effective refractive index if they operate in a higher frequency range [39], the undesired resonances for R-mode (0.7–1.0 THz) will not appear if the amplitude and phase spectra for T-mode (1.0–1.3 THz) are smooth curves. To avoid undesired resonances, the multipole decomposition (Supplementary Section 2) of meta-atoms operating in T-mode under different lattice constant is thoroughly investigated, which have geometric parameters of *a* = 72 μm, *b* = 25 μm, *h*_*v*_ = 5 μm, and *h* = 245 μm. The polarization conversion ratio (PCR) and phase spectra varied with lattice constant of meta-atom are depicted in Figure 3(a)–(c) by red and blue curves, respectively. It can be clearly observed that sharp resonances are excited and they will gradually appear in the concerned frequency range (yellow parts) as the lattice constant increases. These sharp resonances are marked as points A_1_ (1.38 THz), B_1_ (1.49 THz), C_1_ (1.24 THz), D_1_ (1.38 THz), E_1_ (1.17 THz), F_1_ (1.28 THz), and G_1_ (1.37 THz), which are also painted with blue color in Figure 3(d)–(f). The sharp resonances will not only deteriorate the PCR efficiency but also introduce abrupt phase shift near the resonances according to Kramers–Kronig relation [40], which are verified by the points A_2_, B_2_, C_2_, D_2_, E_2_, F_2_, and G_2_. The inferior PCR efficiency will eventually decrease the focusing efficiency of constructed metasurface, and the abrupt phase shifts inevitably make horrible impacts on broadband operation. Moreover, transmission and reflection amplitude of the meta-atoms are also calculated for comprehensive understanding, as depicted in Supplementary Section 2.

As depicted in Figure 3(d)–(f), the sharp peaks from A_1_, B_1_, C_1_, D_1_, E_1_, and F_1_ are primarily attributed to toroidal dipole (TD) resonances, and magnetic dipole (MD) resonance is responsible for G_1_. For instance, there are two magnetic fields with opposite circulating direction in *y*–*z* plane at A_1_, as depicted in left part of Figure 3(g). Therefore, the circulating magnetic dipoles are excited in *x*–*y* plane and eventually induce TD resonance, which accords with the theoretical results of multipole decomposition. Moreover, it can be noticed that the scattering power of magnetic quadrupole is also increased around A_1_, as shown in Figure 3(d). And its magnetic field is depicted in right part of Figure 3(g), which is a parasitical response from coherent oscillation between TD resonance and suppressed electric dipole resonance [41], [42]. From Figure 3(h)–(l), the circulating magnetic fields are appeared in *x*–*y* plane for B_1_, C_1_, and E_1_, while the circulating magnetic fields of D_1_ and F_1_ are excited in *x*–*z* plane, and those circulating magnetic fields are responsible for TD resonances similar to A_1_. Different from the others, G_1_ is induced by MD resonance as shown in Figure 3(f), which is verified by the magnetic fields depicted in Figure 3(m). Furthermore, the *h* of meta-atom also has evident impacts to its performances, as depicted in Supplementary Section 3. According to the investigation, the lattice constant and structural height of meta-atom are eventually set as $p=75/\sqrt{3}$ μm and *h* = 235 μm to suppress undesired resonances, which are thoroughly explained in Supplementary Section 4.

### 3.2 DNN construction and inverse design of TRARM

**Figure 4:**
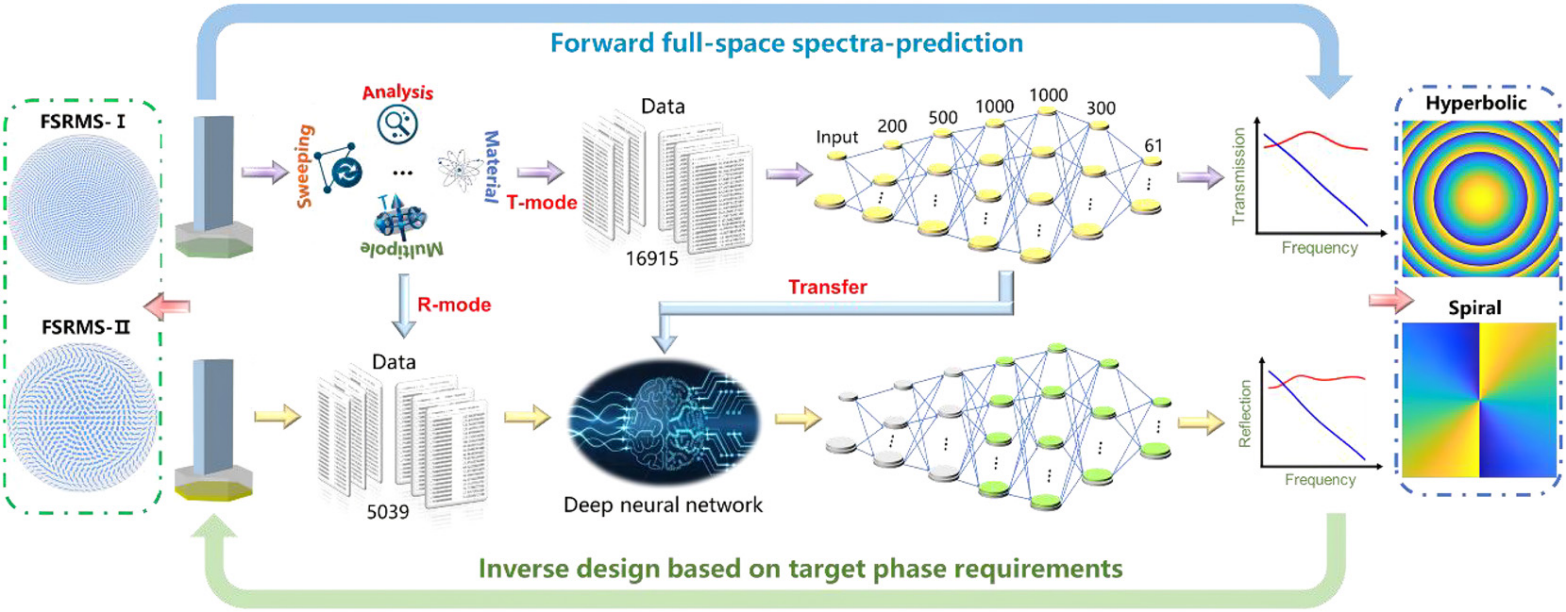
The illustration of design method. Yellow circles are trained neurons, gray circles are transferred neurons, and green circles represent the neurons operating in R-mode.

Firstly, T-mode operating meta-atoms are trained to learn the connections between meta-atoms and their EM responses. Subsequently, transfer learning, permitting trained modes to share their acquired knowledge and experience to improve the performances in other similar scenarios [43], [44], is introduced to decrease the samples from 16,915 data down to 5,039 data. It spent about one month on collecting 16,915 data for T-mode training, while it only took about one week on collecting 5,039 data for R-mode training. As depicted in Figure 4, forward prediction network (FPN) consists of six fully-connected hidden-layers with 200, 500, 1,000, 1,000, 300, and 61 neurons, respectively, and each output layer passes through a ReLU activation function before it is sent to next layer. Besides, the learning rate is set as 10^−4^, and the batch size is 256. Moreover, the weight decay is employed to alleviate the overfitting and ensure the accuracy of the network for test dataset [45].

**Figure 5:**
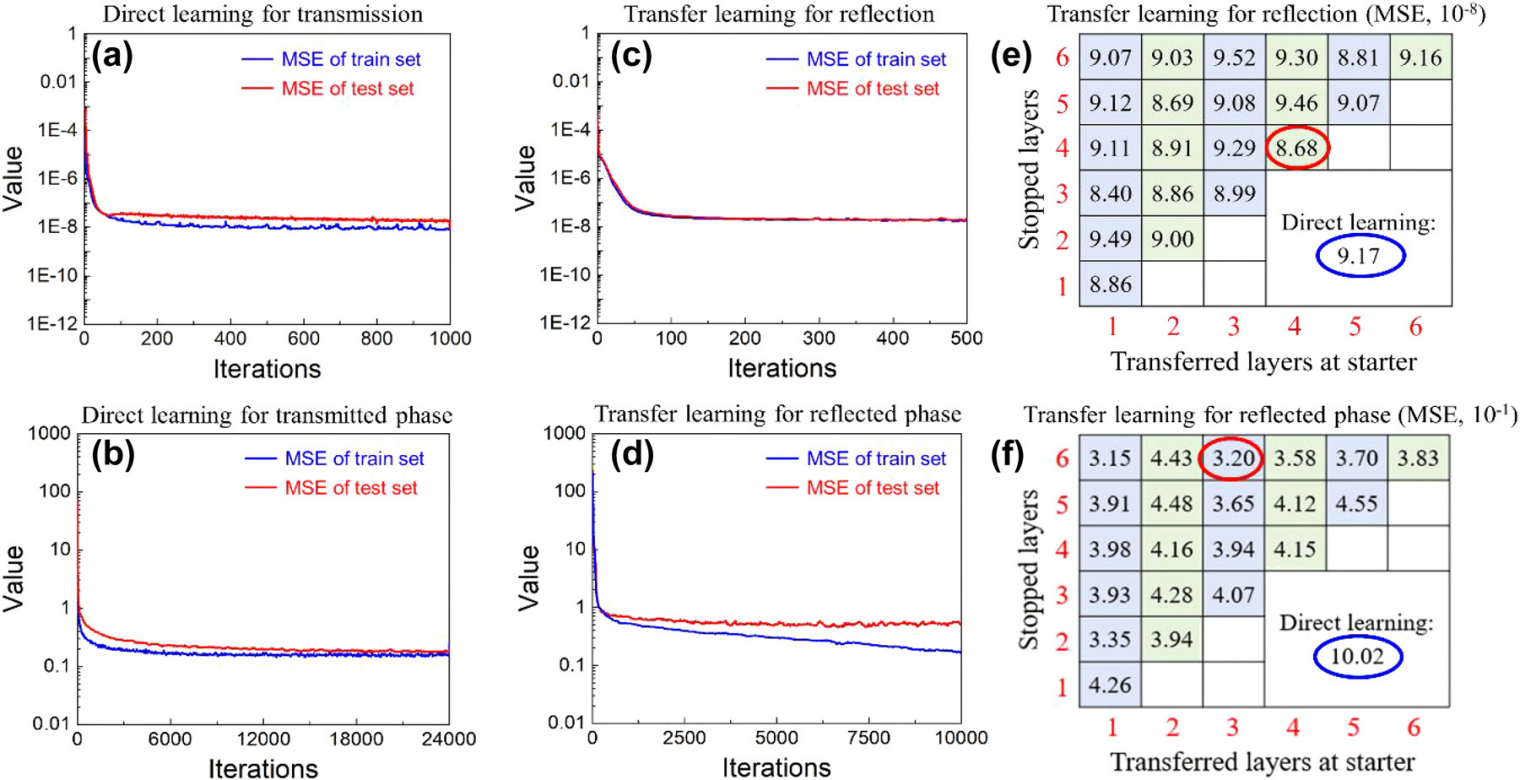
Performances of DNN and transfer learning. (a)–(b) Learning curves of T-mode by direct learning. (c)–(d) Learning curves of R-mode by transfer learning. (e)–(f) MSE of reflected responses of various transferred layers. Red circles are optimal MSE for transfer learning, and blue circles are MSE for direct learning.

It should point out that the PCR efficiencies from established dataset (T-mode meta-atoms) range from 0.23 to 0.99 and the phase responses range from −400.65° to 179.99°, which are depicted in Supplementary Section 5. Therefore, the prediction of PCR efficiency by FPN is much easier than that of phase responses, and it can achieve high accuracy with only 1,000 iterations. The mean square error (MSE) of the PCR efficiency predicted by FPN decreases down to 7.41 × 10^−9^ with around 100 s for the whole training process, as shown in Figure 5(a). Moreover, the transmitted phase response predicted by FPN is set as 24,000 iterations since the large range of variation inevitably requires more iterations to acquire relatively high accuracy, which has the MSE of 2.46 × 10^−1^ and it takes about 65 min for the whole training process, as shown in Figure 5(b). After the direct learning, transfer learning is employed to copy several layers as initialized weights and biases for the training of reflected PCR efficiency and phase response, which have MSE of 8.68 × 10^−8^ (500 iterations) and 3.20 × 10^−1^ (10,000 iterations), respectively, as depicted in Figure 5(c)–(d). The MSE of reflected responses of various transferred layers are also investigated, as shown in Figure 5(e)–(f). Similarly, reflected PCR efficiencies are much easier for prediction than reflected phase responses due to the small variation range.

Moreover, the fractional errors of transmitted PCR efficiency and phase response by direct learning are 5.47 × 10^−6^ and 3.08 × 10^−4^, respectively. And that of reflected PCR efficiency and phase response employing transfer learning are 2.24 × 10^−5^ and 2.34 × 10^−4^, respectively. Such high accuracy demonstrates that the fundamental physical investigations are extremely essential to provide high-quality dataset for DNN training and it can offer a solid foundation to inversely design multifunctional metasurfaces. The comparison between predicted results by FPN and simulated results from finite-integrated method (FIM) are shown in Supplementary Section 6, indicating that the EM responses of various meta-atoms can be precisely predicted by FPN, and it only take a few milliseconds for each meta-atom.

Eventually, the trained FPN can be regarded as an EM simulator to replace conventional simulation methods. In the inverse design, a closed loop design network is employed to satisfy the various phase requirements [31], and its network schematic as well as the training curve of inverse network are depicted in Supplementary Section 7. The inverse design of TRARM can be considered as several steps described as follows. (Ⅰ) The objective metasurface is decomposed into multiple pixels following design principle discussed in Section 2. (Ⅱ) For each pixel corresponding to a target phase, the trained FPN can be activated to predict the EM responses of the meta-atoms, and their parameters are not restricted to the established datasets. (Ⅲ) The output parameters (*a* and *b*) obtained by the counter-propagation of DNN and multi-regression will be fed back to the FPN. It is supposed to provide a new design to further minimize the divergences between predicted results and target phase profiles. (Ⅳ) The previous step will be constantly implemented until the minimum error is achieved, and then output *a* and *b*. Moreover, the meta-atom with higher PCR efficiency will be selected if there are several structures with identical phase profile. (V) The designed metasurface can be eventually acquired by properly arranging the output meta-atoms.

It is also important to note that the proposed high accuracy inverse design method can also be employed to efficiently design the metasurfaces with similar functionalities operating in other frequency range (e.g., visible, infrared, and so on) by appropriately engineering the feature size of corresponding meta-atoms. Moreover, the established high-quality dataset can be regarded as training data and reused in similar cases.

## 4 Results and discussion of TRARM

### 4.1 The performances of TRARM-Ⅰ

**Figure 6:**
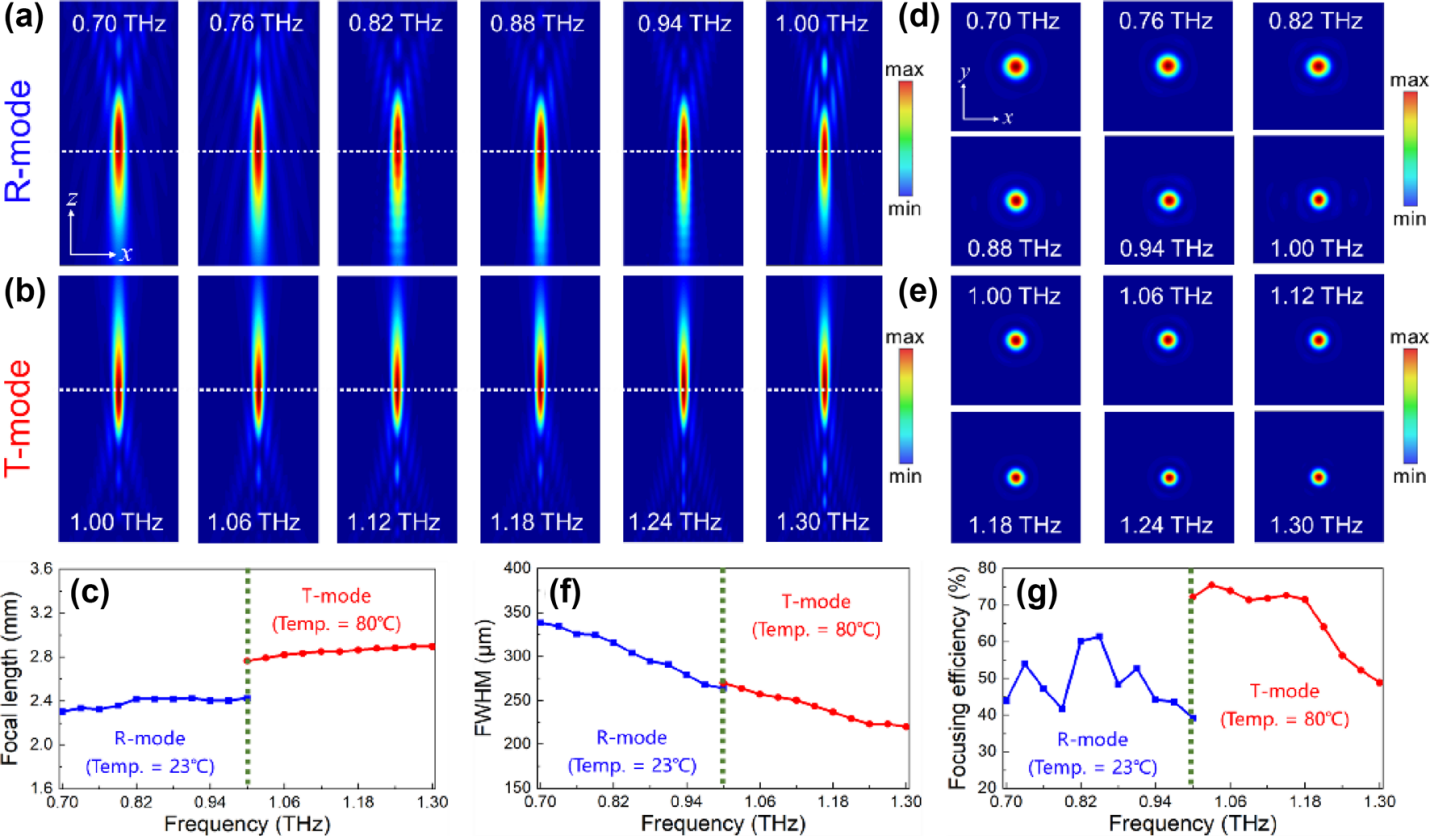
Achromatic performances of TRARM-Ⅰ. (a)–(b) Intensities of TRARM-Ⅰ for R-mode and T-mode in *x*–*z* plane. (c) Corresponding focal length. (d)–(e) Focal plane of TRARM-Ⅰ for R-mode and T-mode. (f)–(g) Corresponding values of FWHM and focusing efficiency.

The phase profiles and configuration of TRARM-Ⅰ, which can be well compensated by the output meta-atoms from the DNN, are shown in Supplementary Section 8. The intensity profiles of reflected and transmitted THz waves in propagation plane of TRARM-Ⅰ are depicted in Figure 6(a) and (b). It is clear that the focal lengths of TRARM-Ⅰ are barely deviated in both modes, indicating that TRARM-Ⅰ is capable of eliminating chromatic aberration in broadband range of 0.7–1.0 THz (R-mode) and 1.0–1.3 THz (T-mode). Moreover, the focal lengths are plotted in Figure 6(c), where the blue and red lines correspond to TRARM-Ⅰ under working temperature of 23 °C and 80 °C, respectively. The average focal length (*F*_*la*_) of TRARM-Ⅰ in R-mode is 2.39 mm and that of T-mode is 2.78 mm, indicating that their corresponding numerical aperture (NA) are 0.58 and 0.52, respectively. The maximum deviation ratios of focal length are 5.14 % (R-mode) and 4.30 % (T-mode). The maximum deviation ratio is described as 100 % × (*F*_lmax_ – *F*_lmin_)/*F*_*la*_, where *F*_lmax_ and *F*_lmin_ indicate the maximum and minimum values of focal length, respectively.

To comprehensively characterize the performances of converged THz waves, the point spread functions (PSFs) of TRARM-Ⅰ are also calculated and depicted in Figure 6(d) and (e). The bright focal spots without any diffusions can be evidently observed in both modes, which further confirms the transmission-reflection-integrated achromatic functionalities of TRARM-Ⅰ. Their intensities are exhibiting a typical Gaussian-type distributions along *x*-direction, as depicted in Supplementary Section 9. The focusing efficiency, which is one of the most crucial criteria to evaluate the performances of achromatic metasurfaces, is also considered and it is defined as 100 % × *I*_*f*_/*I*_in_, where *I*_*f*_ and *I*_in_ represent optical power of focal spot (with a radius of 3 × FWHM, Full Width at Half Maximum) and incident THz waves, respectively [46]. The values of FWHM under R-mode and T-mode are depicted in Figure 6(f). As shown in Figure 6(g), the focusing efficiency of R-mode ranges from 39.27 % at 1.00 THz to 61.47 % at 0.85 THz (blue dotted-line), and that of TRARM-Ⅰ in T-mode ranges from 48.87 % at 1.30 THz to 75.52 % at 1.03 THz (red dotted-line). And the average focusing efficiencies of R-mode and T-mode are 48.79 % and 66.45 %, respectively.

**Figure 7:**
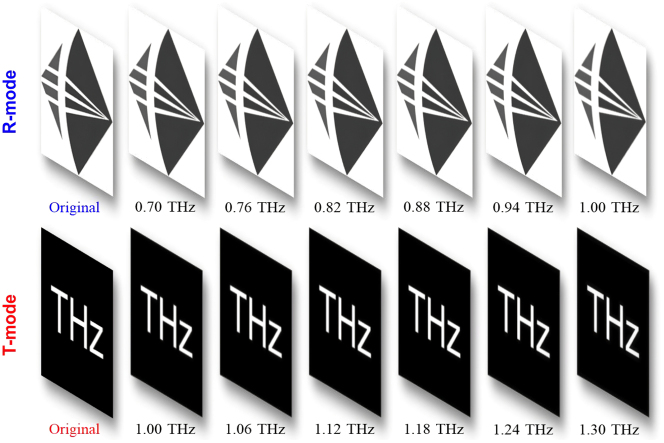
The transmission-reflection-integrated achromatic THz imaging by TRARM-Ⅰ.

The convolution calculations are also performed to demonstrate the capabilities of transmission-reflection-integrated THz imaging based on TRARM-Ⅰ, described as *o*(*x*, *y*) = *g*(*x*, *y*) ⊗ *p*(*x*, *y*), where ⊗ indicates the convolution operation, *o*(*x*, *y*) is the output function at imaging plane, *g*(*x*, *y*) is the complex amplitude of the object, and *p*(*x*, *y*) represents the PSF [10]. In this case, the objects for R-mode and T-mode are chosen as the logo of our institution (grayscale, 1,596 × 1,680 pixels) and the character of “THz” (522 × 522 pixels), respectively. The calculation results are shown in Figure 7, the transmission-reflection-integrated achromatic THz imaging can be well accomplished by TRARM-Ⅰ and the dispersions are barely observed from imaged picture. Moreover, their peak signal-to-noise ratio are discussed in Supplementary Section 10.

### 4.2 The performances of TRARM-Ⅱ

**Figure 8:**
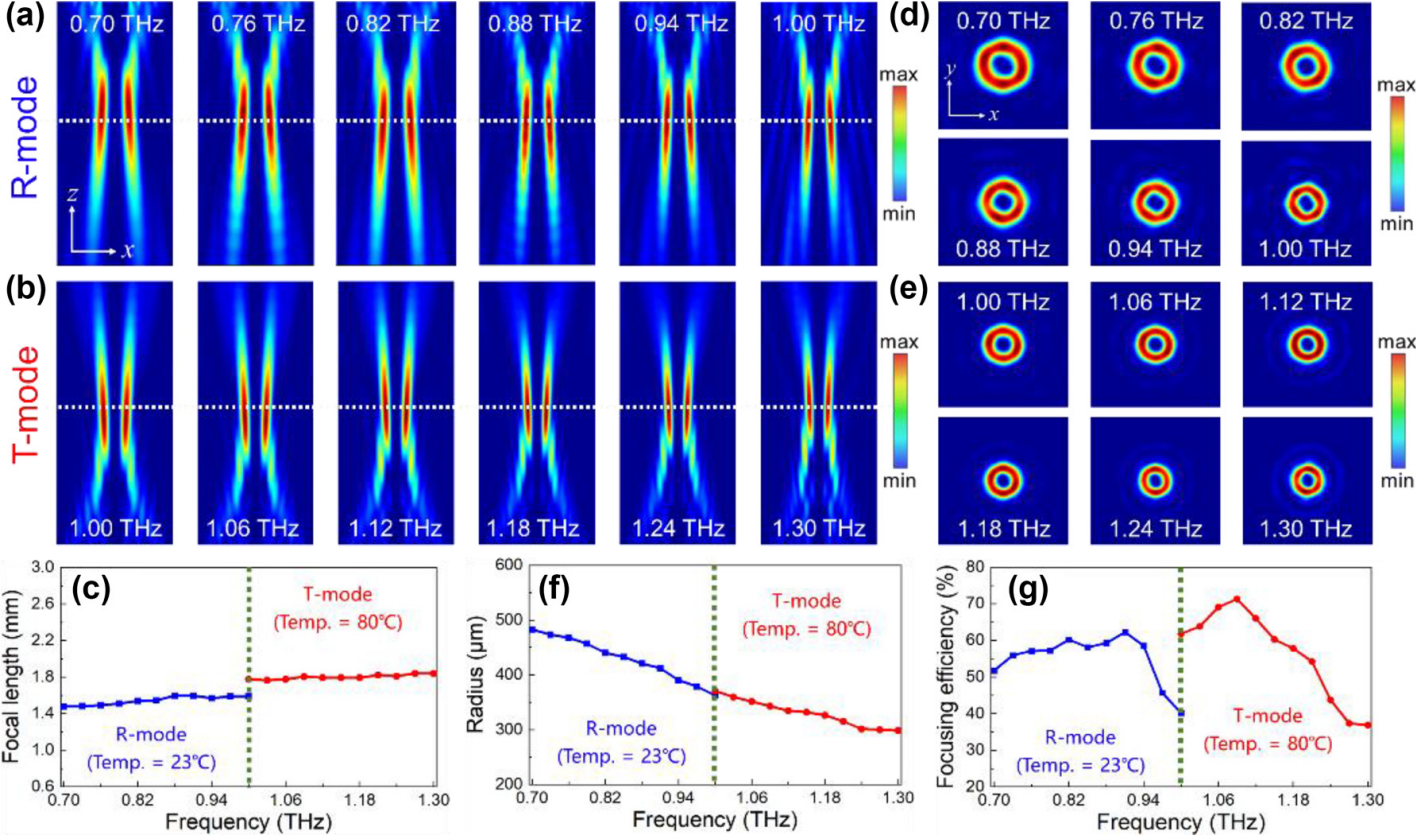
Achromatic performances of TRARM-Ⅱ. (a)–(b) Intensities of TRARM-Ⅱ for R-mode and T-mode in *x*–*z* plane. (c) Corresponding focal length. (d)–(e) Focal plane of TRARM-Ⅱ for R-mode and T-mode. (f)–(g) Corresponding values of FWHM and focusing efficiency.

The phase profiles and configuration of TRARM-Ⅱ are depicted in Supplementary Section 11, and it can be perfectly matched by output meta-atoms from the DNN. It can be clearly observed that the focal length of all the sampled frequency points ranging from 0.7–1.0 THz (R-mode) to 1.0–1.3 THz (T-mode) are nearly constant in Figure 8(a) and (b), implying that TRARM-Ⅱ can correct chromatic aberration in the concerned frequency range and generate FVB in both T-mode and R-mode. To quantitatively elucidate the achromatic performances, the focal lengths of TRARM-Ⅱ are plotted in Figure 8(c). The *F*_*la*_ of TRARM-Ⅱ in R-mode is 1.54 mm and that of T-mode is 1.79 mm, indicating that the NA of two operation modes is 0.67 and 0.62, respectively. And the maximum deviation ratios of focal length are 7.73 % (R-mode) and 4.32 % (T-mode), respectively.

After the exploration of focal length, intensity distributions of focal plane can be subsequently acquired and depicted in Figure 8(d) and (e). Different from the Gaussian-type focal points from TRARM-Ⅰ, the shapes of focal points from TRARM-Ⅱ are similar to doughnuts. The doughnut-shaped distributions manifest the existence of FVB accompanied with a null-amplitude region at focal plane center, which can be also verified by their normalized intensity distributions along *x*-direction (Supplementary Section 12). In addition, the focusing efficiency of generated FVB are also calculated for evaluation, defined as the ratio of optical power inside doughnut ring to that of incident waves [47]. Figure 8(f) shows the radii of doughnut rings of R-mode and T-mode. As shown in Figure 8(g), the focusing efficiencies of TRARM-Ⅱ in R-mode range from 40.18 % at 1.00 THz to 62.26 % at 0.91 THz (blue dotted-line), and that of T-mode range from 36.89 % at 1.30 THz to 71.34 % at 1.09 THz (red dotted-line). Their average focusing efficiencies are 55.14 % (R-mode) and 56.60 % (T-mode), respectively.

**Figure 9:**
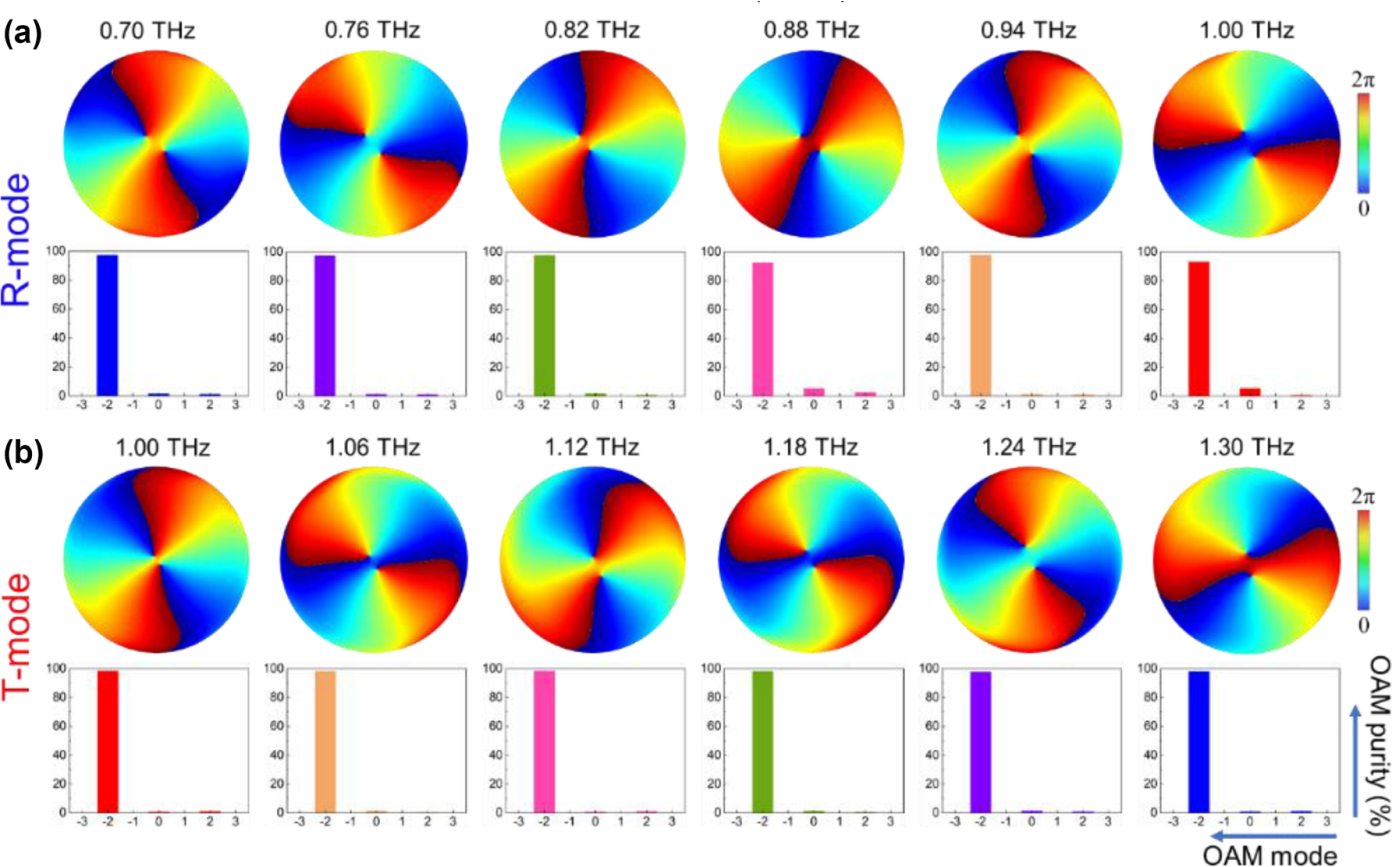
Phase distributions and mode purity of TRARM-Ⅱ at focal point.

Moreover, the phase profiles of focal points are shown in Figure 9(a) and (b). It can be clearly observed that the phase distributions of focal points from TRARM-Ⅱ are all carrying an OAM with *l* = −2 in both R-mode (0.7–1.0 THz) and T-mode (1.0–1.3 THz) according to the spiral branches. Moreover, the calculated values of mode purity are all higher than 92 % at *l* = −2 with average value of 97.02 %. Although there are several parasitic phase noises appeared at *l* = 0 or *l* = +2, they can be neglected compared to the operation mode of *l* = −2.

**Figure 10:**
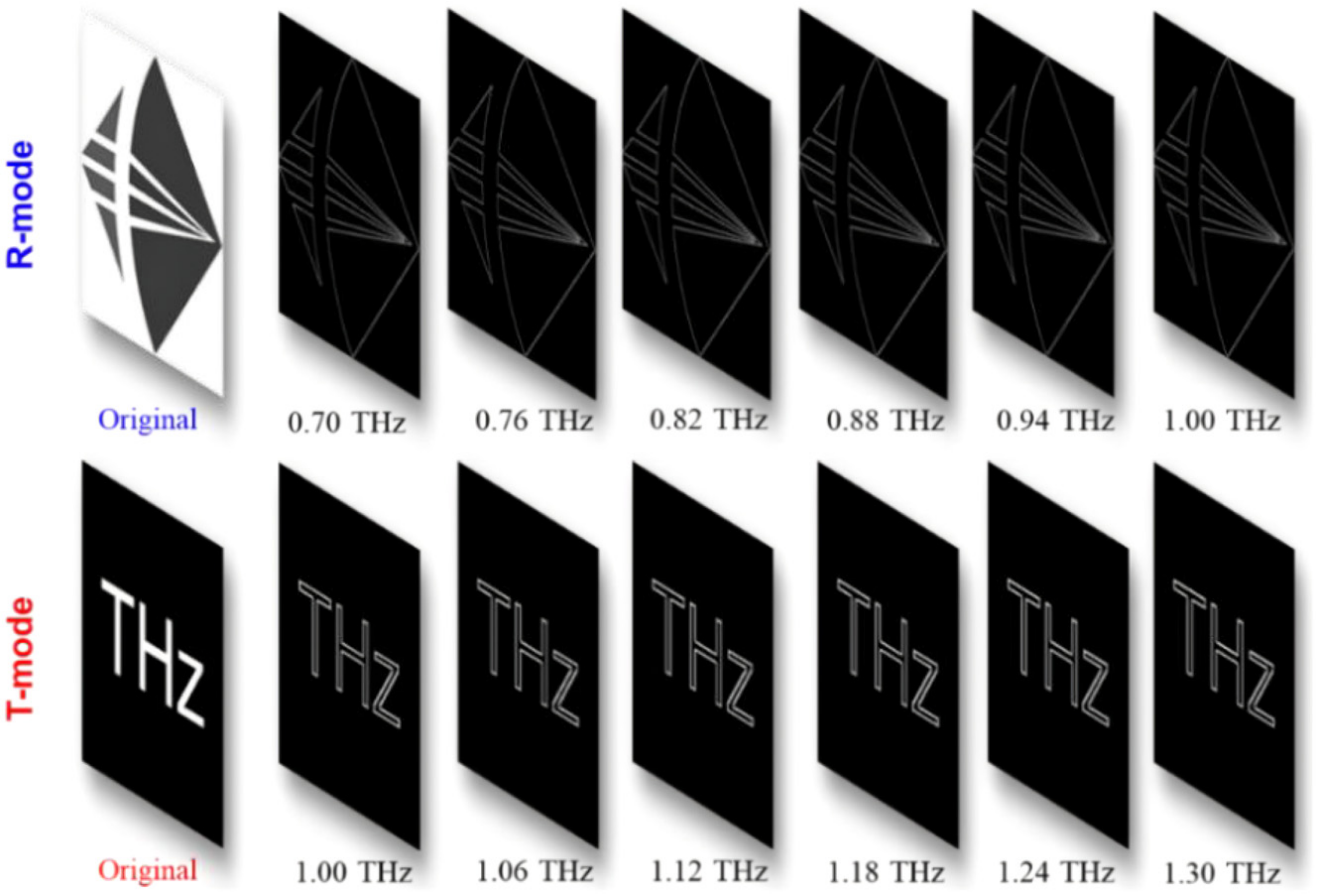
The transmission-reflection-integrated achromatic THz imaging by TRARM-Ⅱ.

As one of the most promising optical methods for edge detection, SPC is a 4-f imaging system employing a vortex beam plate to filter the spatial-frequency signals [11]. Here, the transmission-reflection-integrated edge detections performed by TRARM-Ⅱ associated with *l* = −2 are investigated via convolution operation [48], which can facilitate the integration and miniaturization of SPC system. Same objects are employed for calculation. As shown in Figure 10, the edge information of the input images can be well accomplished in both R-mode and T-mode. Their root-mean-square errors are discussed in Supplementary Section 13. Results indicate that edge information from input images can be well recovered by TRARM-Ⅱ.

It is worth to point out that the TRARM is composed of three phase profiles that greatly increase the design difficulty. And the improved phase design method discussed in Eqs. (2)–(5) requires excellent optical responses of meta-atoms to provide sufficient phase compensation with relatively high reflection (0.7–1.0 THz) and transmission (1.0–1.3 THz). Therefore, DNN is employed to recognize the in-depth connections between geometric parameters and optical responses of metasurfaces, and eventually achieve transmission-reflection-integrated achromatic performances. Moreover, it can be noted that the focusing efficiency of the T-mode is higher than that of the R-mode for both TRARM-Ⅰ and TRARM-Ⅱ, which can be attributed to different optical losses of VO_2_ under different states (metallic phase, M-VO_2;_ and insulator phase, I-VO_2_). In specific, the M-VO_2_ (80 °C) has relative dielectric constant of *ε* = –314.47 + *i*316.25 at 1.0 THz, while relative dielectric constant of I-VO_2_ (23 °C) is *ε* = 9 + *i*8.96 at 1.0 THz. It can be clearly observed that the imaginary part of M-VO_2_ is much higher than that of I-VO_2_, indicating M-VO_2_ has stronger absorption of THz waves. Therefore, the focusing efficiency of the T-mode is higher than that of the R-mode in both TRARM-Ⅰ and TRARM-Ⅱ.

In general, different from the current state-of-the-art techniques that highly depend on algorithm itself and require a large amount of training data as well as testing time, the proposed high accuracy inverse design method in this paper concentrate more on the interaction mechanisms between meta-atoms and incident waves. The physical analyses can provide a high-quality dataset to dramatically decrease the complexity of DNN and significantly improve the prediction accuracy. Therefore, a simple deep learning network with relatively small amount data is enough for the design of advanced meta-devices instead of complicated algorithms. To elucidate this, the clear comparison between state-of-the-art techniques for the inverse design of phase-modulation metasurfaces and the unique benefits of our approach are discussed in Supplementary Section 14.

## 5 Conclusions

In summary, an efficient scheme with high accuracy is proposed to conceive TRARM, which can accomplish achromatic performances while generating FVB in both T-mode and R-mode. The interaction mechanisms of meta-atoms and THz waves are investigated to obtain high-quality dataset. Moreover, transfer learning is employed in design scheme to reduce the quantity requirements of training data by DNN. The training results indicate that the fractional errors of predicted results are less than 10^−4^ for predicting PCR efficiencies and phase responses from FPN. Based on the accurate design scheme and improved design principles, two transmission-reflection-integrated achromatic metasurfaces with different parameters are constructed accordingly. Furthermore, transmission-reflection-integrated achromatic THz imaging and edge detection are also accomplished by the metasurfaces via convolution operation. The results demonstrate that the physical analyses are extremely crucial for the inverse design method, which are able to tremendously simplify the DNNs and greatly improve the prediction accuracy. This work provides a high accuracy inverse design method for conceiving multifunctional meta-devices, which may promise further progress for on-chip THz imaging systems.

## 6 Methods

### 6.1 Calculation of meta-atoms

The FIM is implemented for simulation, and the periodic boundaries are employed in transversal directions and open boundaries are considered in longitudinal axis. The refractive index of Si and SiO_2_ are *n*_Si_ = 3.48 and *n*_SiO2_ = 1.94 [49], [50]. Moreover, the temperature-dependent dielectric permittivity of VO_2_ is discussed in Supplementary Section 15. Moreover, the related references on VO_2_-based tunable THz metasurfaces have been discussed and compared in Table S4.

### 6.2 Data collection

After the theoretical consideration of *p* and *h* of meta-atoms, a dataset of T-mode operating meta-atoms is collected by employing FIM under LCP incident THz waves ranging from 1.0 THz to 1.3 THz with sampling frequency points of *N* = 61. The spectral responses of each meta-atom are determined by *a* and *b*, and their sweeping ranges are *a* ∈ [46.0 μm, 74.0 μm] and *b* ∈ [10.0 μm, 42.0 μm] with a total of 16,915 data (including 80 % training data and 20 % testing data). Since the substrate is a hexagonal structure, the meta-atoms should abide by certain order of $\sqrt{3}ab-75b<1,406.25$ . The selected sweeping range and restricted condition are explained in Supplementary Section 16. In general, the FPN for T-mode meta-atoms has two input parameters (*a*, *b*) associated with the labels of transmitted PCR efficiency *t* (*t*_1_, *t*_2_, …, *t*_61_) and phase profiles *p*_t_ (*p*_t1_, *p*_t2_, …, *p*_t61_), and it has 61 outputs for each prediction. As for R-mode operating meta-atoms, the FPN also has two input parameters (*a*, *b*) with the labels of reflected PCR efficiency *r* (*r*_1_, *r*_2_, …, *r*_61_) and phase profiles *p*_r_ (*p*_r1_, *p*_r2_, …, *p*_r61_), and it also has 61 outputs for predicting each meta-atom of R-mode, which has a total of 5,039 data (including 80 % training data and 20 % testing data) in the identical sweeping range of *a* and *b*. In this case, PyTorch is employed for the experiment, operated on a system with a CPU of Intel Core i9-13900K and an NVIDIA GeForce GTX 4060Ti GPU. And it took about one and a half months for the training data collection, and it spent about an hour and a half for the four sets of data training.

### 6.3 Loss function and error

The loss function of MSE is defined to characterize the FPN, which can evaluate the squared differences between prediction results by FPN (*S*_prediction_) and simulated results by FIM (*S*_FIM_) [31]:

(6) $$\text{MSE}=\frac{1}{N}\underset{i=1,2,\ldots ,N}{\sum }{\left(S_{\text{prediction}}-S_{\text{FIM}}\right)}^{2}$$

The fractional error (FE) is also employed to evaluate the differences between simulated results by FIM and prediction results by FPN [31]:

(7) $$\text{FE}=\frac{1}{N}\underset{i=1,2,\ldots ,N}{\sum }\frac{\left(S_{\text{prediction}}-S_{\text{FIM}}\right)}{S_{\text{FIM}}}$$

Moreover, the comprehensive details regarding the weight decay implementation, including its mathematical formulation within the loss function and the specific value are described in Supplementary Section 18. Besides, the thorough discussion and illustration of the transfer learning process are described in Supplementary Section 19.

### 6.4 Evaluation of TRARM

In order to validate the reliability of proposed inverse design method, two FVB generators with different parameters marked as TRARM-Ⅰ (*l* = 0) and TRARM-Ⅱ (*l* = −2) are rapidly constructed (the corresponding structural parameters of the meta-atoms are listed in Supplementary Section 17). The FIM and vectorial ray-based diffraction integral (VRBDI) are both employed to characterize their performances. To begin with, the FIM is utilized to calculate the near-field EM distributions of TRARM, where the open boundary is applied around the cubic simulation area. In addition, electric boundary is applied at the back of VO_2_ layer for R-mode. Subsequently, VRBDI is employed to further evaluate their far-field performances, which can simultaneously consider ray tracing and Fourier optics [51]. After the calculation of PSF simulated by FIM, the convolution operation can be performed to obtain THz imaging and edge detection. Moreover, in order to demonstrate the merits of the proposed method, the comparison of THz achromatic metasurfaces is depicted in Supplementary Section 20.

### 6.5 Mode purity of OAM

The mode purity is investigated to characterize the performances of FVB generated from TRARM-Ⅱ, which is performed by employing modal decomposition in Fourier transform [16]:

(8) $$\varphi \left(\theta \right)=\overset{+\infty }{\underset{-\infty }{\sum }}\left[\frac{1}{2\pi }\overset{2\pi }{\underset{0}{\int }}\varphi \left(\theta \right)\cdot {\text{e}}^{-\text{i}l\theta }\text{d}\theta \right]\cdot {\text{e}}^{\text{i}l\theta }$$

where *φ*(*θ*) is the phase samples around the doughnut-shaped annulus, and e^i*lθ*^ represents the harmonic item connected with OAM eigenstate.

## Supplementary Material

Supplementary Material Details
