# Supplementary material for: High accuracy inverse design of reconfigurable metasurfaces with transmission-reflection-integrated achromatic functionalities
Source: Nanophotonics. 2025 Mar 25;14(7):921–34. doi: 10.1515/nanoph-2024-0680 (PMC11980877; doi:10.1515/nanoph-2024-0680)
Supplement: Supplementary file 1 — Supplementary Material Details [file j_nanoph-2024-0680_suppl_001.docx]

Supporting Information

High accuracy inverse design of reconfigurable metasurfaces with transmission-reflection-integrated achromatic functionalities achromatic functionalities

Xiao-Qiang Jiang, Wen-Hui Fan,* Xu Chen, Lv-Rong Zhao, Chong Qin, Hui Yan, Qi Wu, and Pei Ju

**Supplementary Section 1: Explanations of selected parameters and frequency range**

There is a trade-off between the parameters of broadband achromatic metasurface. In specific, its operating frequency bandwidth Δ*ω*, maximum achievable radius *R*_max_, and numerical aperture (NA) are restricted by the dispersion range provided by meta-atoms ΔΦ′, which can be described as:^[1]^

$R_{\max}\leq\frac{\Delta\Phi^{'}c}{\Delta\omega\left(\frac{1}{NA}-\sqrt{\left(\frac{1}{NA}\right)^{2}-1}\right)}$ (S1)

where *c* indicates the speed of light in vacuum. In general, the parameters of achromatic metasurfaces are basically depended on the dispersion range provided by meta-atoms.

In this case, two metasurfaces with different parameters are constructed to verify the performances of proposed inverse design method, and their phase requirements can be satisfied by the established dataset. Moreover, the frequency bandwidth, diameter, and focal length of achromatic metasurfaces can be arbitrarily designed if their phase requirements can be satisfied by the meta-atom library. In addition, since the frequency range of 0.7~1.3 THz contains plentiful fingerprints of molecules and chemicals, which has great significance in the applications of THz spectrum and imaging.^[2,3]^ Therefore, this frequency range is selected for demonstrating the TRARM.

Although the phase compensation range of established dataset can support the metasurface with wider frequency range, it will inevitably decrease the focusing efficiency. In this case, the bandwidth of 0.3 THz can ensure that the output meta-atoms have polarization conversion ratio (PCR) efficiencies higher than 50% in the concerned frequency range, which will eventually improve the focusing efficiency of TRARM.

**Supplementary Section 2: The multipole decomposition and the amplitude of meta-atoms**

The scattering powers from different multipoles are defined as:^[4,5]^

$I\text{=}\frac{2\omega^{4}}{3c^{3}}\left|P\right|^{2}\text{+}\frac{2\omega^{4}}{3c^{3}}\left|M\right|^{2}+\frac{2\omega^{6}}{3c^{5}}\left|T\right|^{2}+\frac{\omega^{6}}{5c^{5}}Q_{e}Q_{e}+\frac{\omega^{6}}{20c^{5}}Q_{m}Q_{m}$ (S2)

$P=\frac{1}{i\omega}\int jd^{3}r$ (S3)

$M\text{=}\frac{1}{2c}\int(r\times j) d^{3}r$ (S4)

$T\text{=}\frac{1}{10c}\int\left[(r\cdot j)-2r^{2}j\right] d^{3}r$ (S5)

$Q_{e}=\frac{1}{i\omega}\int\left[r_{\alpha}j_{\beta}+r_{\beta}j_{\alpha}-\frac{2}{3}(r\cdot j)\right] d^{3}r$ (S6)

$Q_{m}=\frac{1}{3c}\int\left[(r\times j)_{\alpha}r_{\beta}+(r\times j)_{\beta}r_{\alpha}\right] d^{3}r$ (S7)

where ***P***, ***M***, ***T***, ***Q_e_***, ***Q_m_***, and ***j*** represent the moments of electric dipole, magnetic dipole, toroidal dipole, electric quadrupole, magnetic quadrupole, and current density respectively.

Moreover, transmission and reflection amplitudes of the meta-atoms mentioned in **Figure 3** are also calculated and depicted in **Figure S1(a)-(f)**.


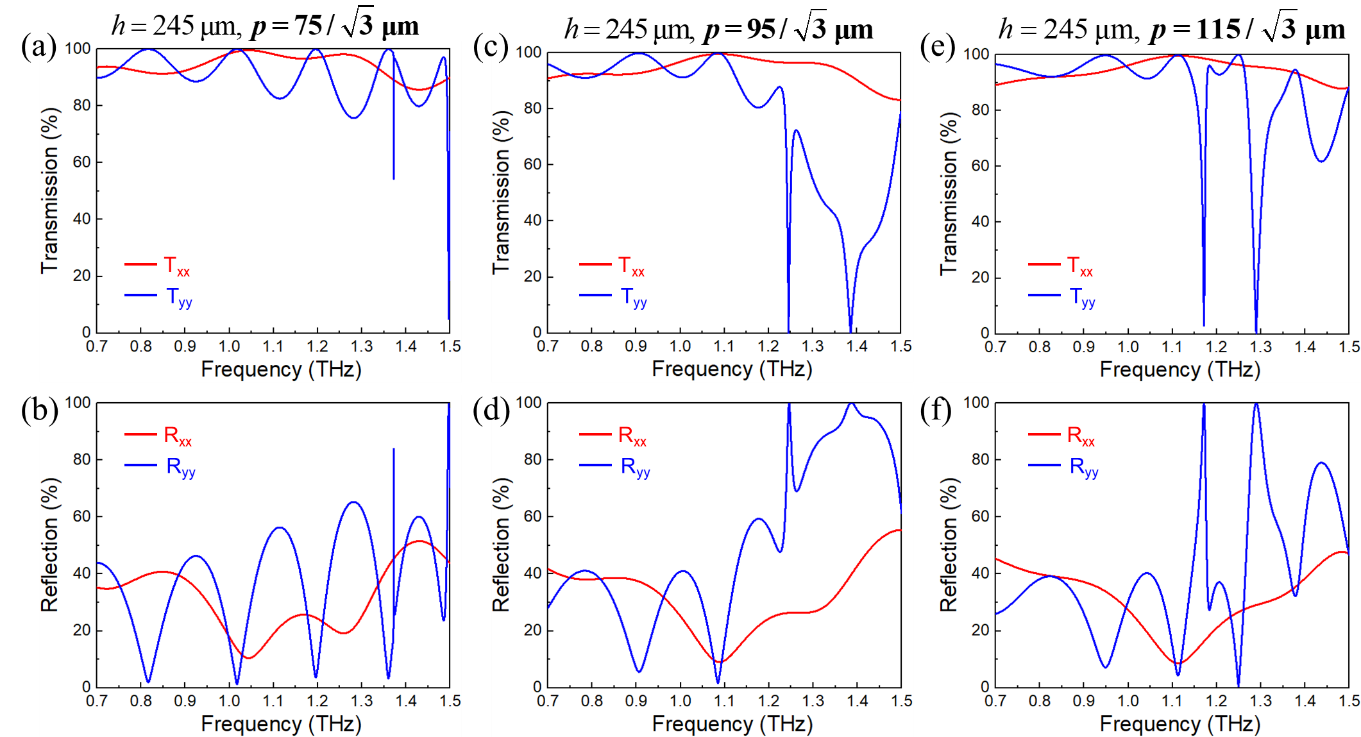


**Figure S1.** Transmission and reflection amplitude of the meta-atoms.

**Supplementary Section 3: The impacts of structural height**

The impacts of structural height *h* are also investigated here, which has the structural parameters of *a* = 72 μm, *b* = 25 μm, *h_v_* = 5 μm, and *p* = $75\text{ }\text{/}\text{ }\sqrt{3}$ μm. For simplicity, only the resonances within the concerned frequency range are investigated. From **Figure S2(a)-(c)**, there are sharp resonances occurred in 1.0~1.3 THz, which are marked as H_1_ (1.30 THz), I_1_ (1.29 THz), and J_1_ (1.28 THz). The sharp resonances inevitably cause the abrupt phase shift near the resonance frequency. To understand their underlying physical mechanism, the multipole decomposition is also performed, which is similar to the lattice constant. From **Figure S2(d)-(f)**, it is clear that the MD and TD are primarily contribute to these undesired resonances. Therefore, the scattering powers of MD and TD are expected to be suppressed by appropriately consider the structural height of meta-atoms.


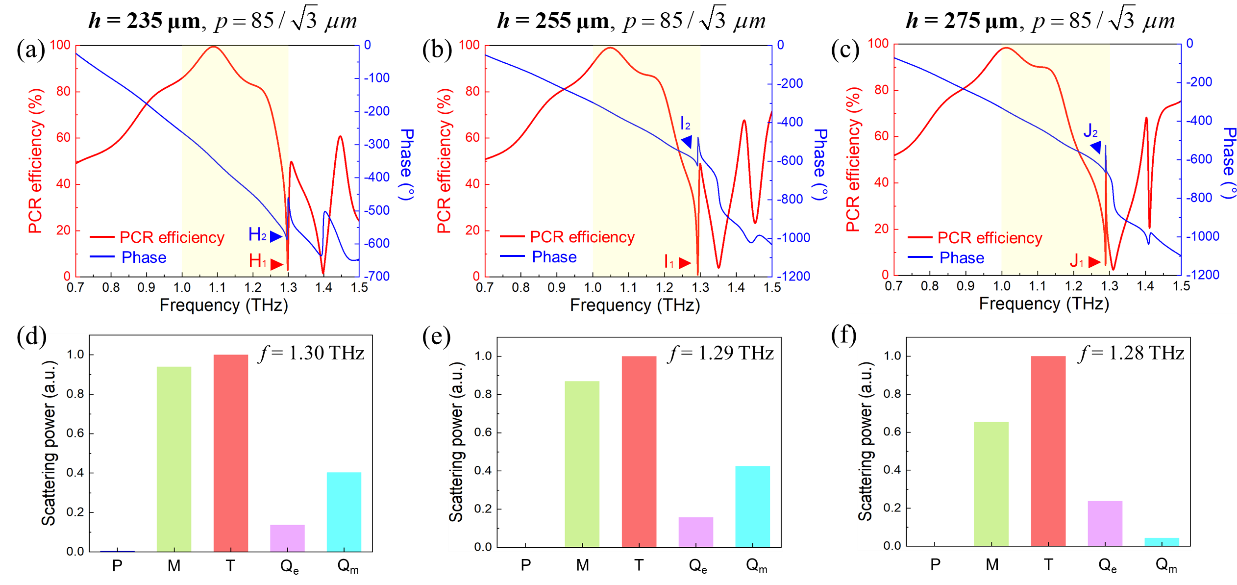


**Figure S2.** (a)-(c) PCR efficiency and phase response of meta-atoms. (d)-(f) Corresponding scattering power of multipole decomposition of three different meta-atoms.

**Supplementary Section 4: The lattice constant and structural height of meta-atoms**

It can be concluded from **Figure 3** and **Figure S2** that the undesired resonances will appear if the lattice constant *p* and structural height *h* of meta-atoms are inappropriately designed. Although the undesired resonances will not be excited if the *p* and *h* of meta-atoms are much smaller than operating wavelength, they cannot provide sufficient phase compensation to realize target functionalities. As discussed in **Section 3.1** from main text, the *p* and *h* of meta-atoms should be as large as possible to provide sufficient phase compensation for transmission-reflection-integrated achromatic operation. Therefore, the lattice constant and structural height of meta-atoms are eventually set as *p* = $75\text{ }\text{/}\text{ }\sqrt{3}$ μm and *h* = 235 μm to suppress the undesired resonances and provide sufficient phase compensation simultaneously.
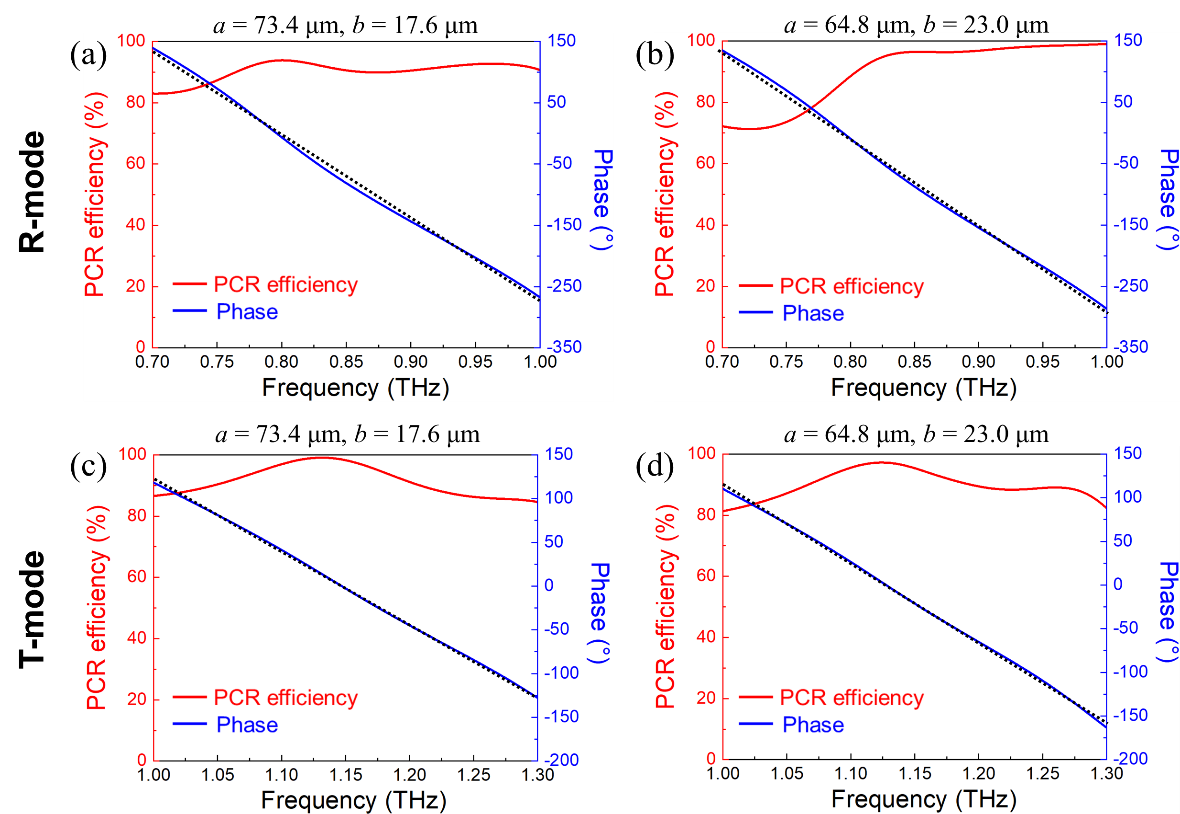


**Figure S3.** The PCR efficiency and phase profiles of meta-atoms.

To elucidate this, the simulated results of two meta-atoms with different *a* and *b* are shown in **Figure S3**. In the case of R-mode operating meta-atoms, the reflected PCR efficiency exhibiting relatively high values (> 70%) without any resonances can be clearly observed in **Figure S3(a)** and **(b)**. Besides, the linear fittings of their phase profiles (black dotted-lines) indicating their *R*-squared values are higher than 0.996, and their fitting functions are *y*_1_ = − 1370.13*x* + 1093.94 and *y*_2_ = − 1424.28*x* + 1131.75, respectively. As for T-mode operating meta-atoms, their transmitted PCR efficiencies are higher than 80% and the *R*-squared values of the phase profiles are greater than 0.999, as shown in **Figure S3(c)** and **(d)**. The fitting functions are *y*_3_ = − 827.72*x* + 949.63 and *y*_4_ = − 905.32*x* + 1020.36, respectively. The meta-atoms with relatively high PCR efficiencies and linear phase responses can provide a solid foundation to achieve TRARM with attractive performances.

**Supplementary Section 5: Numerical range of established dataset for T-mode meta-atoms**


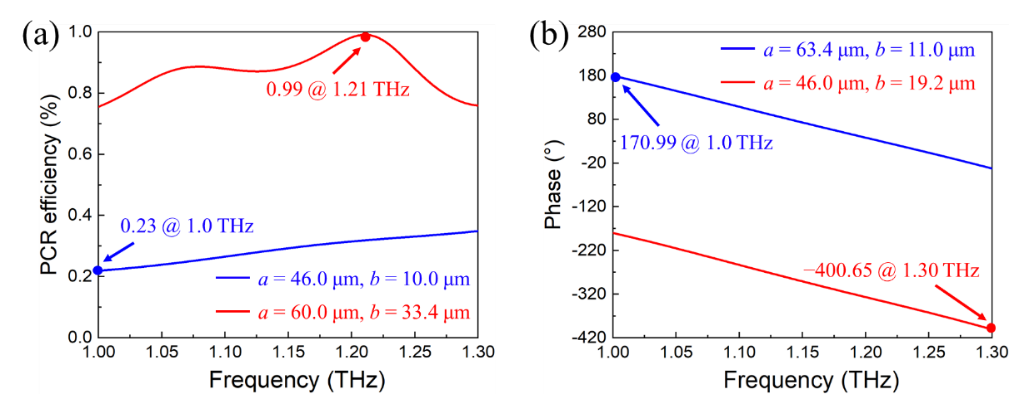


**Figure S4.** The meta-atoms corresponding to the minimum and maximum values in the meta-library.

**Supplementary Section 6: The demonstration of FPN performances**

In order to better demonstrate the attractive capability of the FPN in predicting EM responses from meta-atoms, three test results from meta-atoms with various parameters of *a* and *b* are depicted in **Figure S5**. It is clear that the predicted results (black and green circles) in both R-mode and T-mode are matching well with the simulated EM spectra by FIM (blue and red curves). The test results confirmed that the FPN is able to precisely predict the EM responses of meta-atoms with varied structural parameters.


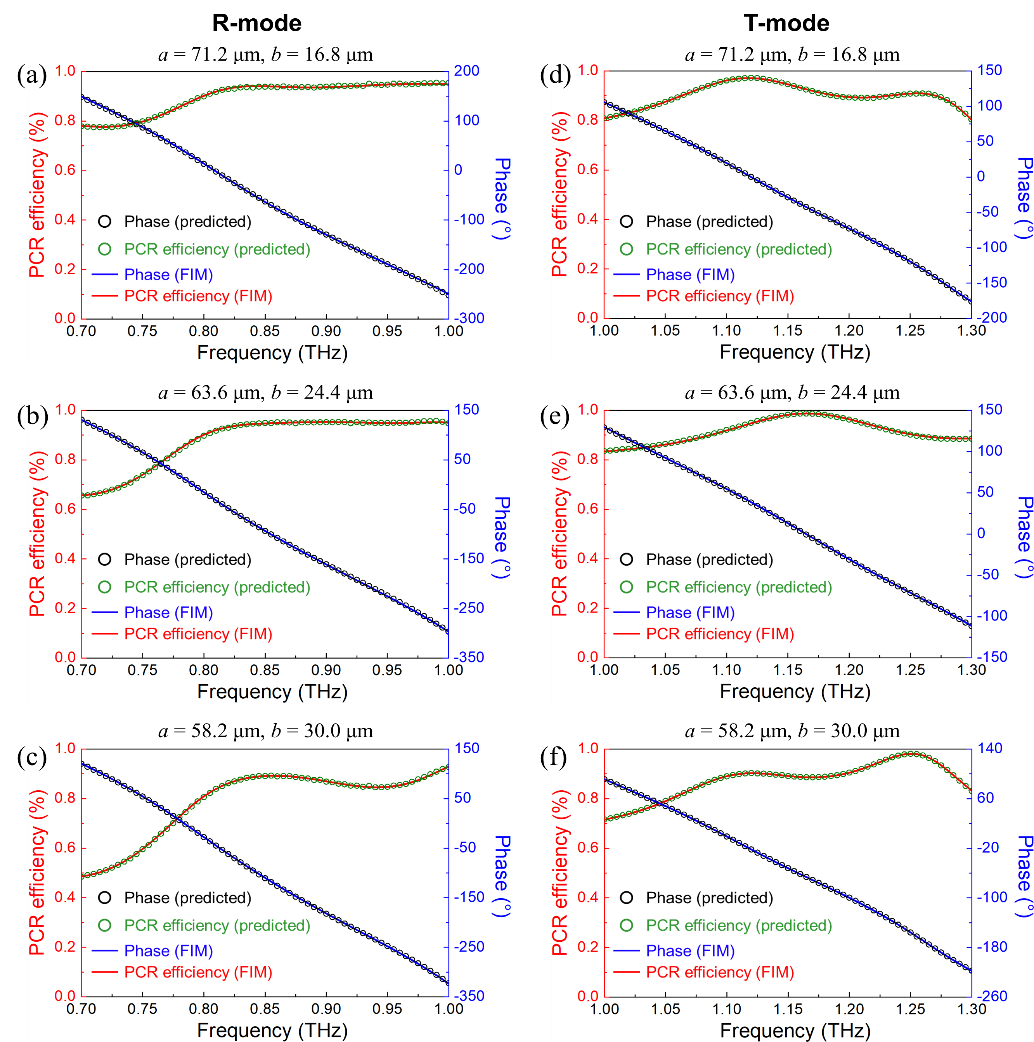


**Figure S5.** (a)-(c) The comparison of FIM and predicted results from R-mode operating meta-atoms. (d)-(f) The comparison of FIM and predicted results from T-mode operating meta-atoms.

**Supplementary Section 7: Schematic of inverse design network**

In the inverse design of the output parameters (*a* and *b*) given by the meta-atom generator will be fed back to the forward prediction network (FPN), and the predicted transmission and phase spectra of the current design are then sent back to the meta-atom generator, where a new design is given to further reduce the difference between the current phase and the target phase, as depicted in Figure 1. And the details are described as follows.


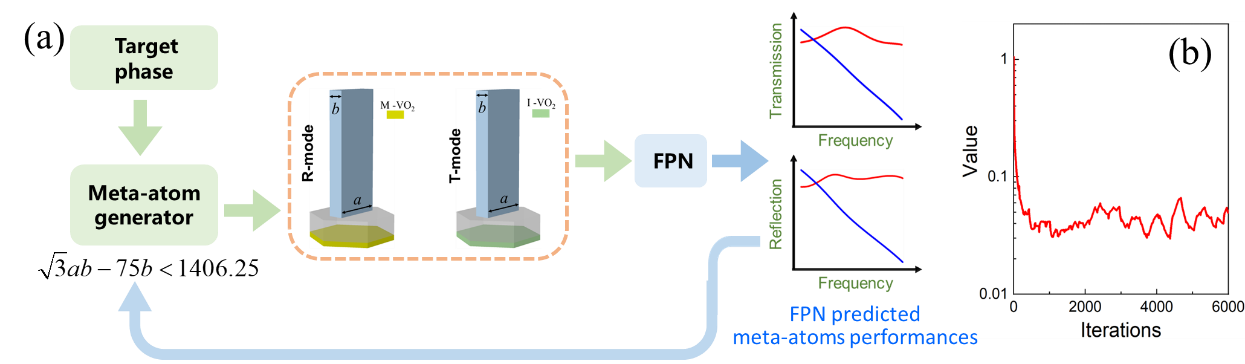


**Figure S6.** (a) Schematic of the closed loop design network. (b) Training curve of inverse network

1. The mechanism of feedback implementation to the neural networks

The feedback mechanism in the proposed neural networks is implemented through backpropagation after the FPN, which involves the following steps:

1. Loss Calculation: A loss function of MSE (mean square error) is defined to evaluate the discrepancy between predictions and ground truth, and the custom loss is also introduced to enforce additional constraints on predictions.
2. Backpropagation: The Chain Rule is employed to calculate the gradient losses with respect to each parameter. And the gradient losses can indicate how to adjust the parameters for minimizing losses.
3. Parameter Update: The updates of model parameters based on gradient losses are conducted by Adam optimizer:

(S8)

where *θ* is the model parameters, ∇*_θ​_L* indicates the gradient losses, and *η* is the learning rate.

1. Iterative Optimization: Repeat FPN, loss calculation, backpropagation, and parameter updates until reaching the designed epochs.
2. Specific parameters being updated to minimize divergences

The closed loop design network consists of six fully-connected hidden-layers with 200, 500, 1000, 1000, 300, and 2 neurons, respectively, and each output layer passes through a ReLU activation function before it is sent to next layer. The custom loss is set as $\sqrt{3}ab-75b$< 1406.25. The learning rate, batch size, and epochs are set as 10^−4^, 256, and 6000 in this work, respectively.

1. Detailed explanation of the multi-regression process and its integration within the overall framework.

Multi-regression can predict multiple continuous outputs simultaneously. In this work, the model predicts two targets (*a* and *b*). And the integration within the overall framework are as follows:

1. Data Preparation: Load input features (reflected phase and transmitted phase profiles) and targets (*a* and *b*), and split into training set and validation set.
2. Training and validation: Train the model on the training set while monitoring validation loss, and save the best model checkpoint based on validation performance by evaluating the total losses (MSE and custom loss).
3. Result Saving: Export loss values and output targets to files.

The framework integrates data preparation, training, and validation to predict two targets effectively.

**Supplementary Section 8: The phase profile and configuration of TRARM-Ⅰ**

As for TRARM-Ⅰ with preset topological charge of *l* = 0, it can be also considered as the achromatic metalens with no spiral phase profile. It has focal length of *F_lr_*_-Ⅰ_ = *F_lt_*_-Ⅰ_ = 2.80 mm and diameter of *D*_1_ = 3.38 mm. According to the design principle and preset parameters, the TRARM-Ⅰ can be constructed by 23 various meta-atoms and each of them can concurrently satisfy the complex phase difference (black and purple circles) for both R-mode (blue curve) and T-mode (red curve), as depicted in **Figure S7(a)**. And its configuration in *x*-*y* plane is illustrated in **Figure S7(b)**. The reconfigurable functionalities of TRARM-Ⅰ can be readily accomplished by altering ambient temperature, which will cause the reversible phase transition of VO_2_ between insulator and metal.


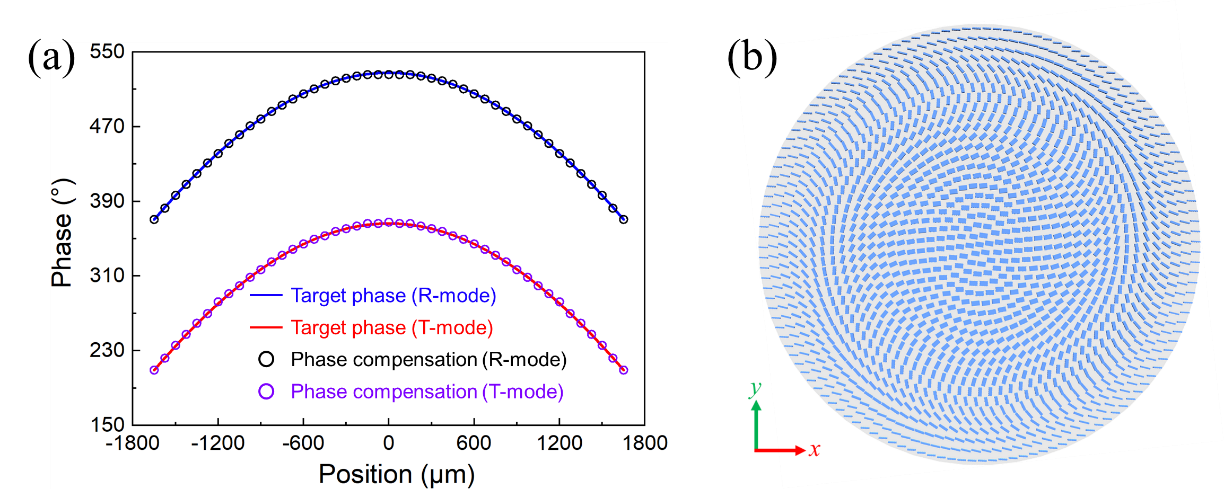


**Figure S7.** (a) Phase profiles of TRARM-Ⅰ. (b) Configuration of TRARM-Ⅰ in *x*-*y* plane.

**Supplementary Section 9: Intensities of TRARM-Ⅰ along *x*-direction in focal plane**


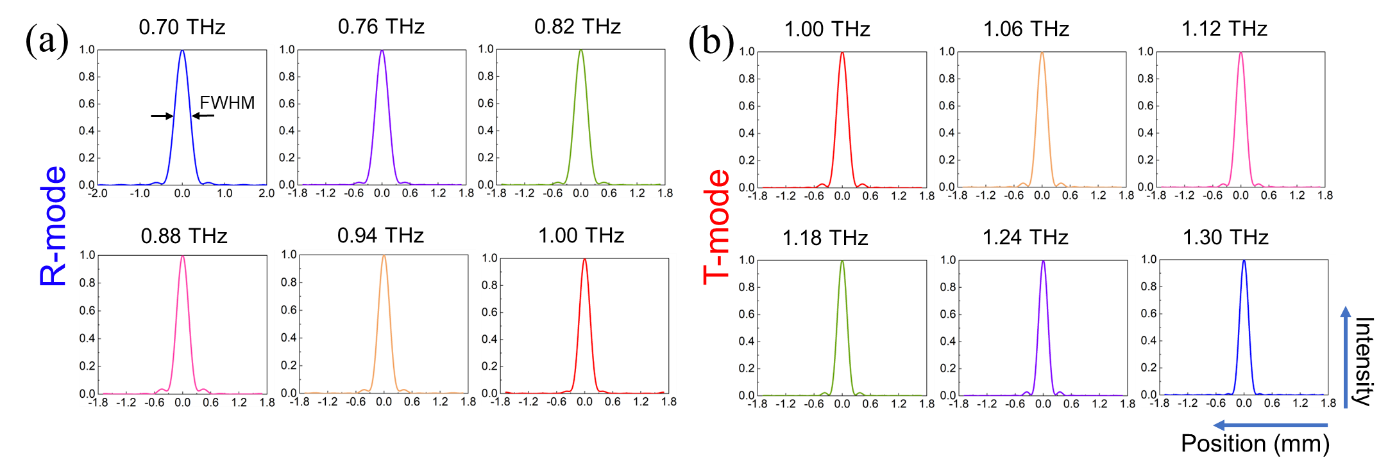


**Figure S8.** Intensity distributions of TRARM-Ⅰ along *x*-direction in focal plane

**Supplementary Section 10: The evaluation of broadband THz imaging by** **TRARM-Ⅰ**

In order to evaluate the performances of TRARM-Ⅰ in the application of THz imaging, the peak signal-to-noise ratio (PSNR) is investigated. The PSNR is one of the most crucial criteria to evaluate the quality of the images, which is defined as:^[6]^

$PSNR=20\cdot\lg\left(\frac{L}{\sqrt{MSE}}\right)$ (S9)

where *L* is the maximum gray value. Moreover, MSE (mean squared error) is the average value of the squared intensity differences of distorted image pixels (*D*) and original image pixels (*O*):

$MSE=\frac{1}{mn}\sum_{i=0}^{m-1} \sum_{j=0}^{n-1} \left[O(i,j)-D(i,j)\right]^{2}$ (S10)

where *m* and *n* indicate the image size. Therefore, the PSNR of THz images from TRARM-Ⅰ can be calculated according to **Equations S9** and **S10**. And the results are listed in **Table S1**, which are all higher than 23 dB and barely changed in the operating frequency range, indicating that TRARM-Ⅰ can be employed for broadband THz imaging.

**Table S1.** The PSNR of the THz images by TRARM-Ⅰ.


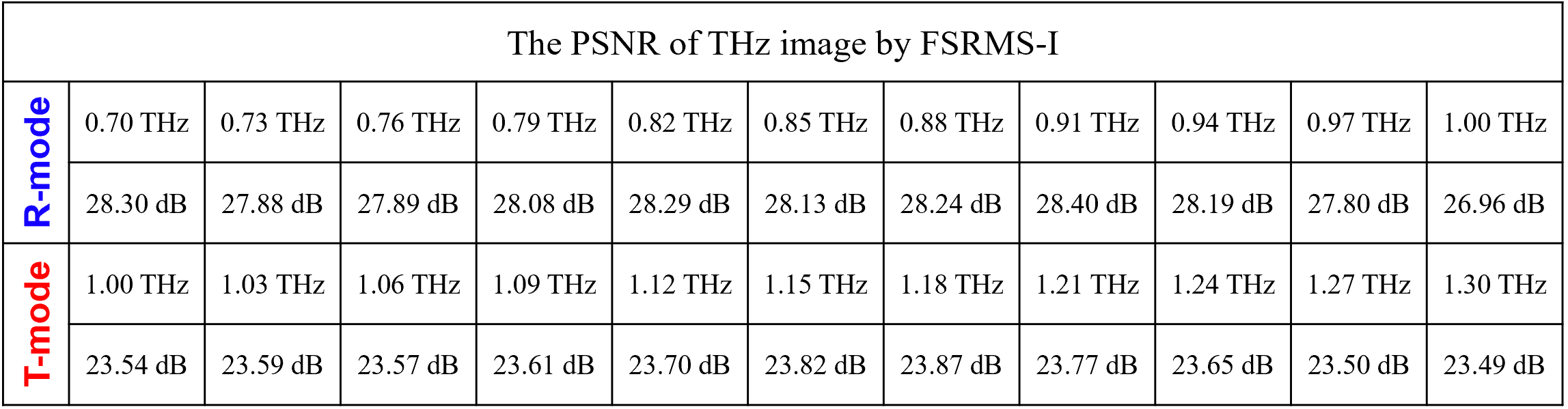


**Supplementary Section 11: The phase profile and configuration of TRARM-Ⅱ**

The focal length and diameter of TRARM-Ⅱ are set as *F_lr_*_-Ⅱ_= *F_lt_*_-Ⅱ_ = 1.80 mm and *D*_2_ = 2.80 mm, and it carries the OAM with topological charge of *l* = –2. From **Figure S9(a)**, the phase compensations of selected meta-atoms (black and purple circles) are able to satisfy the phase requirements of R-mode (blue curve) and T-mode (red curve). The TRARM-Ⅱ constructed by 19 various meta-atoms following target phase profiles is depicted in **Figure S9(a)**.


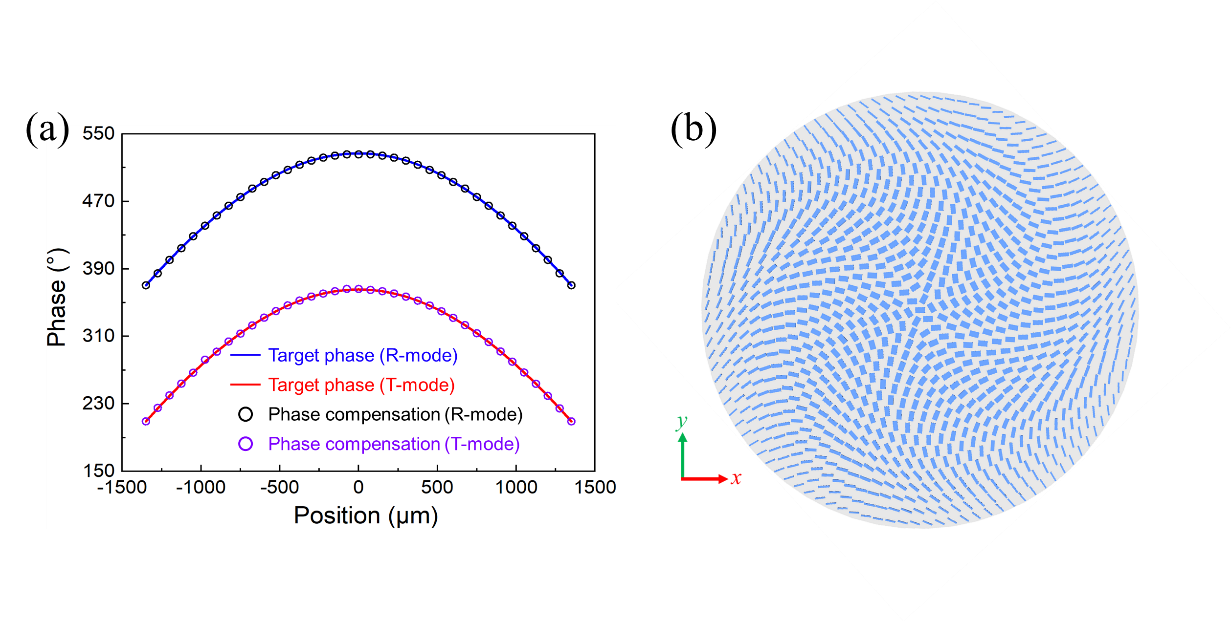


**Figure S9.** (a) Phase profiles of TRARM-Ⅱ. (b) Configuration of TRARM-Ⅱ in *x*-*y* plane.

**Supplementary Section 12: Intensities of TRARM-Ⅱ along *x*-direction in focal plane**


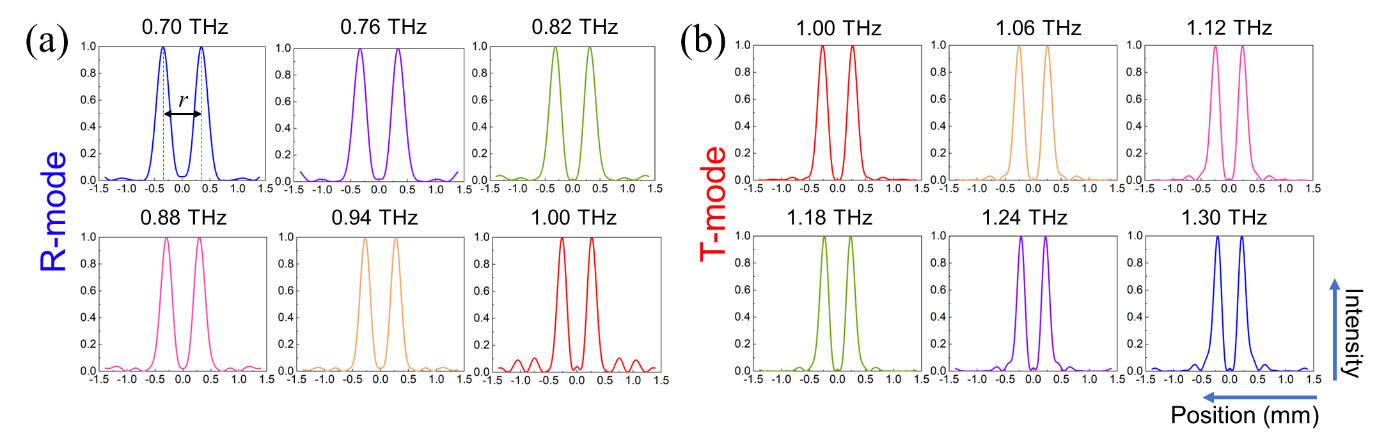


**Figure S10.** Intensity distributions of TRARM-Ⅱ along x-direction in focal plane

**Supplementary Section 13: The evaluation of edge detection by TRARM-Ⅱ**

In order to evaluate the performances of TRARM-Ⅱ in the application of edge detection, the root-mean-square error (RMSE) is also investigated, which is defined as:^[7]^

$RMSE=\sqrt{\frac{\sum_{i=1}^{m} \sum_{j=1}^{n} \left[f_{r}(i,j)-f_{a}(i,j)\right]^{2}}{m\times n}}$ (S11)

where *m* and *n* indicate the image size, *f_r_*(*i*, *j*) and *f_a_*(*i*, *j*) indicate the restructured and the anticipant edge signal of the images.

Therefore, the RMSE of edge detection from TRARM-Ⅱ can be calculated according to **Equation S11**. And their results are listed in **Table S2**. The RMSE of the edge information of the images are less than 0.17 and they are barely changed in the operating frequency range. The results indicate that the edge information from input images can be well recovered in the broadband THz range by TRARM-Ⅱ.

**Table S2.** The RMSE of edge detection by TRARM-Ⅱ.


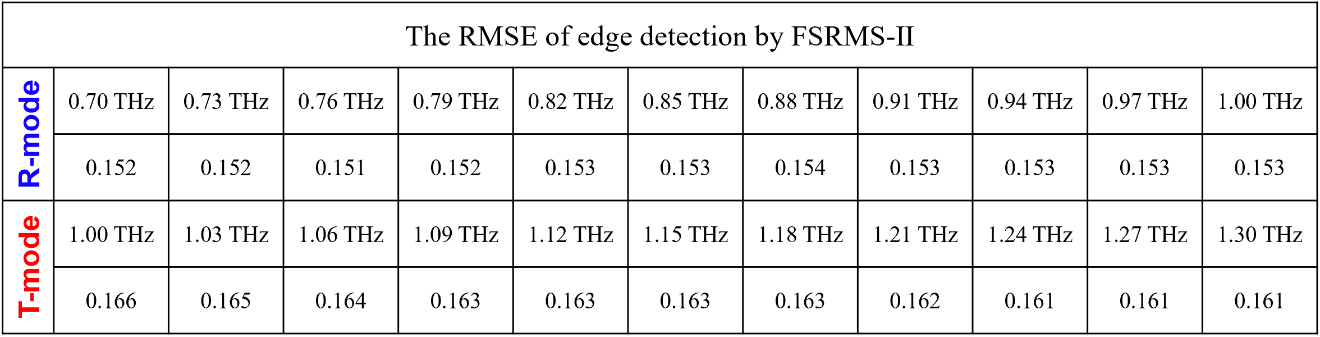


**Supplementary Section 14: The** **comparison between state-of-the-art techniques and proposed high accuracy inverse design method for phase-modulation metasurfaces**

**Table S3.** Recent progress of inverse design method for conceiving phase-modulation metasurfaces.

| Reference | Functionality | Operating range | Training error | Method |
| --- | --- | --- | --- | --- |
| [10] | Phase delay | 430, 540, and  667.5 nm | Average phase  error of 16°  (~10^5^ datasets) | DNN |
| [11] | Meta-filters and reconfigurable metasurfaces | 58 THz | Fractional errors of 15.0% and 24.7%  (35000 datasets) | DNN |
| [12] | Focusing metalens | 57 THz | Average phase error of 0.78°  (~10^5^ datasets) | GAN and PNN |
| [13] | Microwave cloak | 6.8~8.2 GHz | Mean relative error of 1.47%  (10000 datasets) | ANN |
| [14] | Bifocal metalens | 50 THz | phase error of ±10°  (29000 datasets) | GAN |
| [15] | Meta-hologram | 1.5 μm | MSE of phase training is 0.0112  (~10^5^ datasets) | PDNN |
| [16] | Near-infrared  multispectral imaging | 1150, 1350, and  1550 nm | MSE of phase training is 0.003  (20000 datasets) | CNN |
| [17] | Broadband thermal imaging based on meta-optics | 8~12 μm | Training loss of 0.048  (~10^5^ datasets) | DNN |
| This work | transmission-reflection-integrated and achromatic  vortex beam generator | 0.7~1.3 THz | Fraction error less than 10^−4^  (16915 datasets for direct training, 5039 datasets for transfer learning) | Physical analyses  and DNN |

**^DNN^**^: Deep Neural Network^; **^GAN^**^: Generative Adversarial Network^; **^PNN^**^: Predicting Neural Network^; **^ANN^**^: Artificial Neural Network^; **PDNN**: Physics-driven Deep Neural Network; **^CNN^**^: Convolutional Neural Network^.

# **Supplementary Section 15: Dielectric permittivity of VO_2_**

The classical Drude model can be employed to describe the dielectric permittivity of metallic phase of VO_2_ (M-VO_2_) in THz range:^[8]^

$\varepsilon_{m}(\omega)=\varepsilon_{\infty}-\frac{\omega_{p}^{2}}{\omega(1+iq/m^{*}\mu)}$ (S12)

where *ε*_∞_ represents the high-frequency contribution to relative permittivity, *ω* denotes the angular frequency, *m*^*^ is the effective mass of electron, *q* is the electronic charge and *μ* is the electron mobility. Besides, the plasma frequency is defined as *ω_p_* = (*Nq*^2^/*ε*_0_*m*^*^)^1/2^, where *ε*_0_ is the permittivity in vacuum and the electron concentration *N* = 1.3 × 10^28^ m^−3^. ^[8]^

During IMT process, the effective dielectric permittivity *ε_eff_* of VO_2_ can be explained by Bruggeman effective-medium theory:^[9]^

$\varepsilon_{eff}(\omega)=0.25\times\left\{\varepsilon_{i}(2-3f_{m})+\varepsilon_{m}(3f_{m}-1)+\sqrt{[\varepsilon_{i}(2-3f_{m})+\varepsilon_{m}(3f_{m}-1)]^{2}+8\varepsilon_{i}\varepsilon_{m}}\right\}$ (S13)

where *ε_i_* denotes the dielectric permittivity of insulator phase of VO_2_ (I-VO_2_), and *f_m_* indicates the volume fraction of metallic phase:

$f_{m}(T)=0.95\times\left\{1-\frac{1}{1+e^{[(T-T_{0})/\Delta T]}}\right\}$ (S14)

where *T* is the ambient temperature, *T*_0_ = 68 ℃ represents the transition temperature, and ∆*T* = 2 ℃ is the bandwidth of transition temperature. Moreover, the relations among *f_m_*, *ε_m_*, *ε_i_* and *ε_eff_* can be described by effective-medium approximation:^[9]^

$f_{m}\frac{\varepsilon_{m}-\varepsilon_{eff}}{\varepsilon_{m}+(d-1)\varepsilon_{eff}}+(1-f_{m})\frac{\varepsilon_{i}-\varepsilon_{eff}}{\varepsilon_{i}+(d-1)\varepsilon_{eff}}=0$ (S15)

where *d* = 2 is the dimensionality of composite medium.


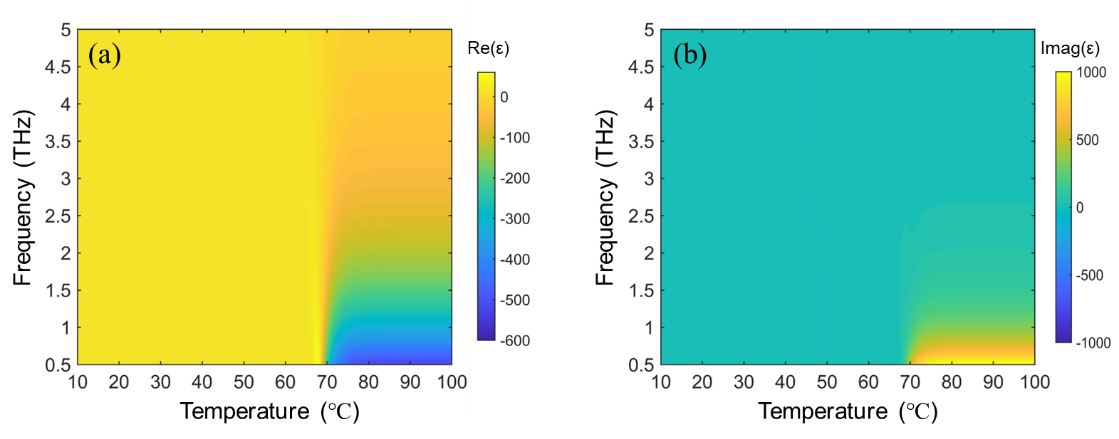


**Figure S11.** Dielectric permittivity of VO_2_. (a) real part, and (b) imaginary part.

Therefore, the dielectric permittivity of VO_2_ under various temperatures can be obtained from **Equations** **(S12) ~ (S15)** and they are depicted in **Figure S11**. At room temperature, it is clear that I-VO_2_ is similar to dielectric materials with negligible absorption losses for transmitting THz waves. On the contrary, M-VO_2_ at thermal environment has evident dielectric losses, which is similar to the metal.

Moreover, the related references on VO_2_-based tunable metasurfaces have been discussed and compared in Table S4.

**Table S4.** Recent progress of VO_2_ metasurfaces for manipulating THz waves.

| Reference | Functionality | Operating frequency | Method |
| --- | --- | --- | --- |
| [18] | Reconfigurable quarter-wave plate | 0.617 THz | Finite-element method |
| [19] | Electrical tunable amplitude modulation | 0.27~0.91 THz | Finite-element method |
| [20] | Optical tunable amplitude modulation | 0.4~1.8 THz | Numerical computation by CST Microwave Studio |
| [21] | Switchable focusing metalens | 0.65 THz | Numerical computation by CST Microwave Studio |
| [22] | Dual-control electromagnetically  induced transparency | 0.39 / 0.72 THz | Numerical computation by CST Microwave Studio |
| [23] | Thermally Controlled Optical Encryption | 0.63 THz | Numerical computation by CST Microwave Studio |
| [24] | Reconfigurable wide-angle beam-steering | 0.6 THz | Numerical computation by CST Microwave Studio |
| This work | transmission-reflection-integrated and achromatic vortex beam generator | 0.7~1.3 THz | Physical analyses  and DNN |

**Supplementary Section 16: The sweeping range and restricted condition of length *a* and width *b* of meta-atom**

In order to obtain relatively high focusing efficiency of TRARM, the meta-atoms with PCR efficiency less than 20% are not considered in our design. Therefore, the sweeping ranges of length *a* and width *b* of meta-atom are set to *b* ∈ [46.0 μm, 74.0 μm] and *b* ∈ [10.0 μm, 42.0 μm].

Moreover, the substrate is a hexagonal structure as depicted in **Figure S12(a)**, and the rectangular meta-atom should locate inside the hexagonal substrate. To meet the requirement, the top-right part is considered for analyses and shown in **Figure S12(b)**. For a right triangle with acute angle of 60°, the area of inside rectangular structure should be less than $\sqrt{3}\times p^{2}/16$. Therefore, the *a* and *b* of meta-atom should satisfy the condition as follows:

$\frac{a-p}{2}\times\frac{b}{2}<\frac{\sqrt{3}\times p^{2}}{16}$ (S16)

By simplifying the **Equation S16,** the certain order of $\sqrt{3}ab-75b<1406.25$ can be eventually obtained.


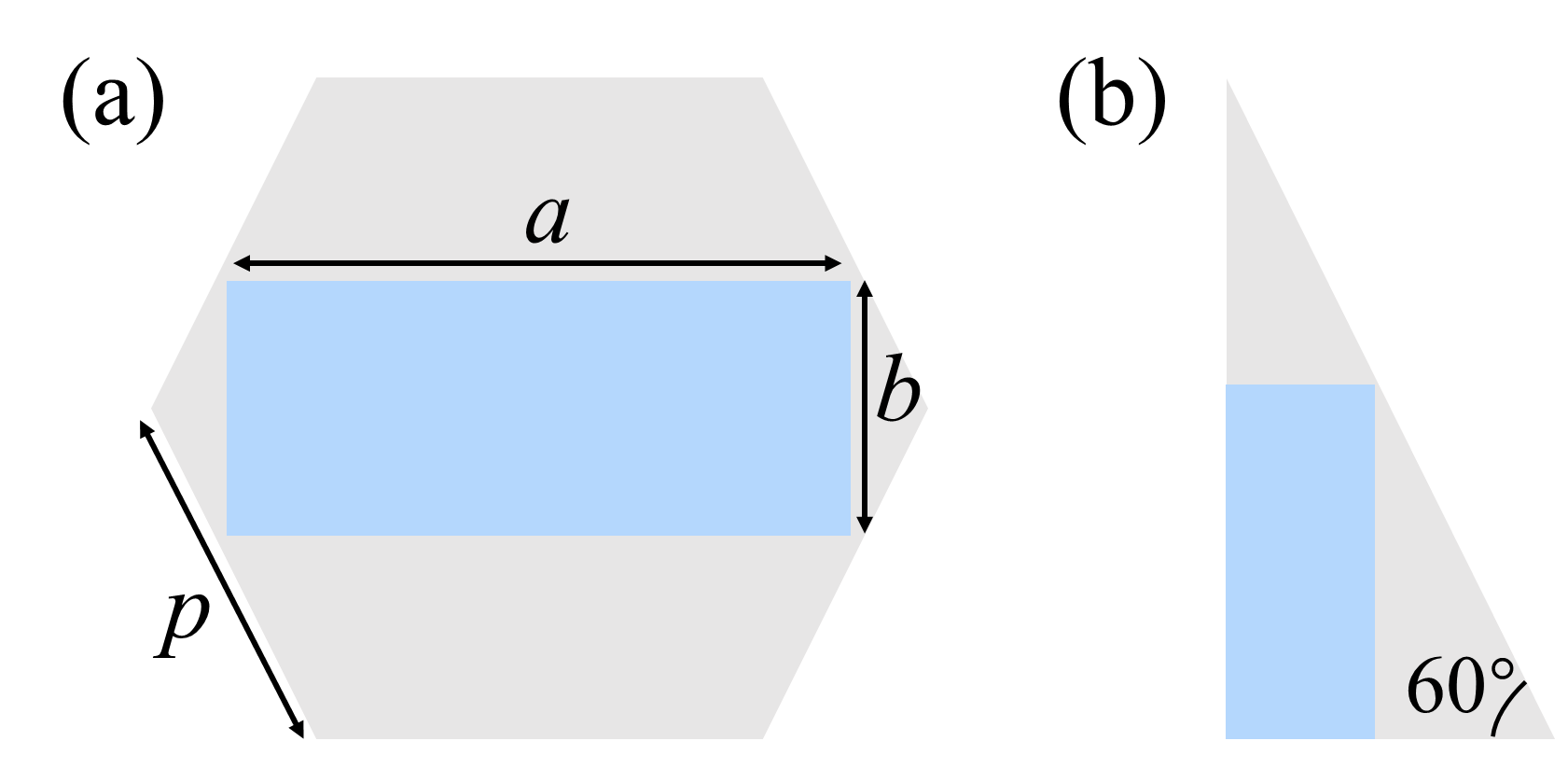


**Figure S12.** (a) The top view of meta-atom. (b) The top-right part of meta-atom.

**Supplementary Section 17: The structural geometries of the meta-atoms**

Considering the accuracy of current deep silicon etching techniques, only one decimal place is retained for length *a* and width *b* of meta-atoms.

**Table S5.** The structural geometries of the meta-atoms in TRARM-Ⅰ.


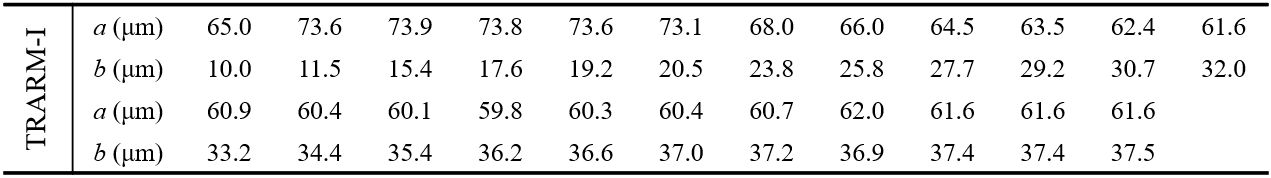


**Table S6.** The structural geometries of the meta-atoms in TRARM-Ⅱ.


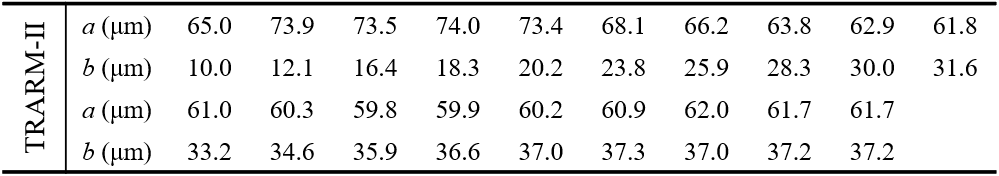


**Supplementary Section 18: The comprehensive details regarding the weight decay**

Weight decay is a regularization technique and it prevents overfitting by adding a penalty term related to the model parameters to the loss function. Specifically, the loss function with weight decay can be expressed as [25]:

(S17)

where *L*_data_ represents the original data loss, *λ* is the regularization coefficient controlling the strength of the regularization term, *R*(**w**) is the regularization term. As for the L2 regularization (weight decay), the regularization term *R*(**w**) is defined as:

(S18)

And the loss function incorporating weight decay can be expressed as:

(S19)

During the optimization process, weight decay encourages the model parameters to gravitate towards smaller values, thereby reducing the model complexity and preventing overfitting.

In this work, the weight decay is implemented within SGD (Stochastic Gradient Descent) optimizer, in which the parameter updates can be expressed as [26]:

(S20)

where ∂*L*_0_ /∂*ω_i_* indicates the original gradient. Therefore, in order to add the L2 regularization to the loss function, the term of *λω_i_* is incorporated into the model gradients during the parameter update. And the parameter specifying the regularization strength for L2 regularization is set as 10^−2^, which determines the penalization of model weights during training to prevent overfitting.

**Supplementary Section 19: The discussion and illustration of the transfer learning**

Transfer learning focuses on improving model performance by reducing the distribution discrepancy between the source and target domains, and it can be described by domain adaptation theory [27]. In specific, source domain *D_s_* and target domain *D_t_* can be described as:

(S21)

The data distributions of *D_s_* and *D_t_* can be considered as *P_s_* (*x*, *y*) and *P_t_* (*x*, *y*), respectively. Assume that the input space *X* and output space *Y* are the same for both domains, but the data distributions are different, i.e., *P_s_* (*x*, *y*) ≠ *P_t_* (*x*, *y*).

The transfer learning can be employed to train the model *h* using data from source domain and a small amount of labeled data from the target domain (*h*: *X* → *Y*), and minimize the generalization error of target domain. The generalization error of target domain can be expressed as:

(S22)

where *ℓ* is the loss function. Since the target domain only has a small amount of labeled data, the data from the source domain is expected to assist the learning processes. Therefore, it is necessary to analyze how the difference between the source and target domains affects the generalization error, which can be measured by using divergence metrics of maximum mean discrepancy (MMD) [28]:

(S23)

where *ϕ* is a feature mapping, and *H_k_*​ is a reproducing kernel Hilbert space.

Assuming the feature distributions of the source and target domains are quite similar, the transfer learning can minimize the following loss function:

(S24)

where *ε_s_*(*h*) is the empirical error on the source domain:

(S25)

Therefore, the MMD in this case can be expressed as:

(S25)

In general, by optimizing the Eq. S25, the source domain error and the distribution discrepancy can be simultaneously reduced, thereby improving the model performances on target domain. The learning rate is set as 10^−4^, and the batch size is 256 in this work.

**Supplementary Section 20: The comparison of THz achromatic metasurfaces**

**Table S7.** The comparison of THz achromatic metasurfaces.

| Reference | Operating range | Functionality | Numerical aperture | Working efficiency |
| --- | --- | --- | --- | --- |
| [29] | 0.3 – 0.8 THz | Achromatic metalens | 0.385 | 68% (Maximum) |
| [30] | 2.29 – 2.70 THz | Sub-diffraction focusing | 0.125 | 14.07 – 72.47% |
| [31] | 0.9 – 1.4 THz | Tunable dispersion | / | 26.1 – 33.9% |
| [32] | 0.4 – 0.8 THz | Airy beam generation | / | 20 – 60% |
| [33] | 100 – 130 μm | Super-resolution Focusing | 0.2846 | 26.87 – 49.84% |
| [34] | 0.5 – 1.1 THz | Achromatic metalens | 0.47 | ~ 43% (Average value) |
| This work | 0.7 – 1.3 THz | Transmission-reflection-integrated and achromatic vortex beam generator | TRARM-Ⅰ: 0.58  TRARM-Ⅱ: 0.52 | TRARM-Ⅰ: 48.79% (R-mode) and 66.45% (T-mode)  TRARM-Ⅱ: 55.14% (R-mode) and 56.60% (T-mode) |

**References**

1. S. Shrestha, A. C. Overvig, M. Lu, A. Stein, N. F. Yu, Broadband achromatic dielectric metalenses, Light-Sci. Appl. 7 (2018) 85.
2. R. N. Zeng, Y. J. Bian, X. Zhang, Z. Q. Zhu, B. Yang, Terahertz spectroscopy study of the stereoisomers of threonine, Appl. Spectrosc. 76 (2017) 1132-1141.
3. H. Yan, W. H. Fan, X. Chen, L. T. Liu, H. Q. Wang, X. Q. Jiang, Terahertz signatures and quantitative analysis of glucose anhydrate and mixture, Spectroc. Acta Pt. A-Molec. Biomolec. Spectr. 258 (2021) 119825.
4. X. Chen, W. H. Fan, Toroidal metasurfaces integrated with microfluidic for terahertz refractive index sensing, J. Phys. D: Appl. Phys. 52 (2019) 485104.
5. 1. V. Savinov, V. A. Fedotov, N. I. Zheludev, Toroidal dipolar excitation and macroscopic electromagnetic properties of metamaterials Phys. Rev. B 89 (2014) 205112.
6. Z. Wang, A. C. Bovik, H. R. Sheikh, E. P. Simoncelli, Image quality assessment: from error visibility to structural similarity, IEEE Trans. Image Process. 13 (2004) 600-612.
7. H. Guo, L. Wang, S. Zhao, Compressed ghost edge imaging Chin. Opt. Lett. 17 (2019) 071101.
8. Z. G. Ban, Y. Shi, N. Q. Huang, L. Li, X. G. Lu, H. F. Zhu, Q. W. Shi, W. X. Huang, T. J. Cui, Modeling terahertz properties of vanadium dioxide by *Ab Initio* computational scheme and its experimental verification, Phys. Rev. Appl. 18 (2022) 064095.
9. P. U. Jepsen, B. M. Fischer, A. Thoman, H. Helm, J. Y. Suh, R. Lopez, R. F. Haglund, Metal-insulator phase transition in a VO_2_ thin film observed with terahertz spectroscopy, Phys. Rev. B 74 (2006) 205103.
10. D. J. Liu, Y. X. Tan, E. Khoram, Z. F. Yu, Training deep neural networks for the inverse design of nanophotonic structures, ACS Photonics 5 (2018) 1365.
11. S. S. An, C. Fowler, B. W. Zheng, M. Y. Shalaginov, H. Tang, H. Li, L. Zhou, J. Ding, A. M. Agarwal, C. Rivero-Baleine, K. A. Richardson, T. Gu, Hu, J. J. H. L. Zhang, A deep learning approach for objective-driven all-dielectric metasurface design, ACS Photonics 6 (2019) 3196-3207.
12. S. S. An, B. W. Zheng, M. Y. Shalaginov, H. Tang, H. Li, L. Zhou, J. Ding, A. M. Agarwal, C. Rivero-Baleine, M. K. Kang, K. A. Richardson, T. Gu, J. J. Hu, C. Fowler, H. L. Zhang, Deep learning modeling approach for metasurfaces with high degrees of freedom, Opt. Express 28 (2020) 31932-31942.
13. C. Qian, B. Zheng, Y. C. Shen, L. Jing, E. P. Li, L. Shen, H. S. Chen, Deep-learning-enabled self-adaptive microwave cloak without human intervention, Nat. Photonics 14 (2020) 383-390.
14. S. S. An, B. W. Zheng, H. Tang, M. Y. Shalaginov, L. Zhou, H. Li, M. K. Kang, K. A. Richardson, T. Gu, J. J. Hu, C. Fowler, H. L. Zhang, Multifunctional metasurface design with a generative adversarial network, Adv. Opt. Mater. 9 (2021) 2001433.
15. W. Wei, P. Tang, J. Z. Shao, J. Zhu, X. Y. Zhao, C. Z. Wu, End-to-end design of metasurface-based complex-amplitude holograms by physics-driven deep neural networks, Nanophotonics 11 (2022) 2921-2929.
16. B. Xiong, Y. H. Xu, W. W. Li, W. Ma, T. Chu, Y. M. Liu, Deep Learning Design for Multiwavelength Infrared Image Sensors Based on Dielectric Freeform Metasurface, Adv. Opt. Mater. 12 (2024) 2302200.
17. L. C. Huang, Z. Y. Han, A. Wirth-Singh, V. Saragadam, S. Mukherjee, J. E. Fröch, Q. A. A. Tanguy, J. Rollag, R. Gibson, J. R. Hendrickson, P. W. C. Hon, O. Kigner, Z. Coppens, K. F. Böhringer, A. Veeraraghavan, A. Majumdar, Broadband thermal imaging using meta-optics, Nat. Commun. 15 (2024) 1662.
18. Y. Nakata, K. Fukawa, T. Nakanishi, Y. Urade, K. Okimura, F. Miyamaru, Reconfigurable terahertz quarter-wave plate for helicity switching based on babinet inversion of an anisotropic checkerboard metasurface, Phys. Rev. Appl. 11 (2019) 044008.
19. Y. Li, H. Ma, Y. Wang, J. Ding, L. M. Qi, Y. L. Fu, R. Ning, L. Rong, D. Y. Wang, X. P. Zhang, Electrically driven active VO_2_/MXene metasurface for the terahertz modulation, Appl. Phys. Lett. 121 (2022) 241902.
20. X. L. Zhao, J. Lou, X. Xu, Y. Yu, G. M. Wang, J. H. Qi, L. X. Zeng, J. He, J. G. Liang, Y. D. Huang, D. P. Zhang, C. Chang, Multifield controlled terahertz modulator based on silicon‐vanadium dioxide hybrid metasurface, Adv. Opt. Mater. 10 (2022) 2102589.
21. W. Kou, W. Q. Shi, Y. X. Zhang, Z. Q. Yang, T. Chen, J. Q. Gu, X. L. Zhang, Q. W. Shi, S. X. Liang, F. Lan, H. X. Zeng, Terahertz switchable focusing planar lens with a nanoscale vanadium dioxide integrated metasurface, IEEE Trans. Terahertz Sci. Technol. 12 (2022), 13-22.
22. H. Wang, Y. T. Zhang, F. R. Hu, M. Z. Jiang, L. H. Zhang, W. T. Zhang, J. G. Han, Active dual-control terahertz electromagnetically induced transparency analog in VO_2_ metasurface, Appl. Phys. Lett. 123 (2023) 061701.
23. S. Q. Zhu, B. W. Dong, G. X. Guo, X. G. Lu, Q. Xu, J. G. Han, W. X. Huang, H. Ma, Y. T. Wang, X. Q. Zhang, L. L. Huang, Terahertz metasurfaces for thermally controlled optical encryption, Laser Photon. Rev. 17 (2023) 2300233.
24. F. Y. Yang, T. C. Tan, S. Prakash, A. Kumar, A. Ariando, R. Singh, N. Wang, P. Pitchappa, Reconfigurable wide-angle beam-steering terahertz metasurfaces based on vanadium dioxide, Adv. Opt. Mater. 12 (2024) 2302047.
25. X. X. Jia, X. C. Feng, H. W. Yong, D. Y. Meng, Weight decay with tailored adam on scale-invariant weights for better generalization, IEEE Trans. Neural Netw. Learn. Syst. 35 (2024) 6936-6947.
26. P. Zhou, X. Y. Xie, Z. C. Lin, K. C. Toh, S. C. Yan, Win: weight-decay-integrated nesterov acceleration for faster network training, J. Mach. Learn. Res. 25 (2024) 83.
27. S. Ben-David, J. Blitzer, K. Crammer, A. Kulesza, F. Pereira, J. W. Vaughan, A theory of learning from different domains, Mach. Learn. 79 (2010): 151-175.
28. Z. H. Peng, W. Zhang, N. Han, X. Z. Fang, P. P. Kang, L. Y. Teng, Active transfer learning, IEEE Trans. Circuits Syst. Video Technol. 30 (2020): 1022-1036.
29. Q. Q. Cheng, M. L. Ma, D. Yu, Z. X. Shen, J. Y. Xie, J. C. Wang, N. X. Xu, H. M. Guo, W. Hu, S. M. Wang, T. Li, S. L. Zhuang, Broadband achromatic metalens in terahertz regime, Sci. Bull. 64 (2019): 1525-1531.
30. F. Zhao, Z. P. Li, X. M. Dai, X. Y. Liao, S. Li, J. C. Cao, Z. G. Shang, Z. H. Zhang, G. F. Liang, G. Chen, H. Li, Z. Q. Wen, Broadband achromatic sub-diffraction focusing by an amplitude-modulated terahertz metalens, Adv. Opt. Mater. 8 (2020): 2000842.
31. Z. X. Shen, S. H. Zhou, X. A. Li, S. J. Ge, P. Chen, W. Hu, Y. Q. Lu, Liquid crystal integrated metalens with tunable chromatic aberration, Adv. Photonics, 2 (2020): 036002.
32. Q. Q. Cheng, J. C. Wang, L. Ma, Z. X. Shen, J. Zhang, X. Y. Zheng, T. Chen, Y. Yu, D. Yu, Q. He, W. Hu, T. Li, S. L. Zhuang, L. Zhou, Achromatic terahertz Airy beam generation with dielectric metasurfaces, Nanophotonics, 10 (2021): 1123-1131.
33. S. Li, Z. P. Li, X. M. Dai, Y. R. Li, X. Y. Liao, J. Cao, Z. Q. Wen, H. Li, G. Chen, Constructing a frequency-dependent phase profile of linear dispersion for achromatic superresolution focusing, Phys. Rev. Appl. 18 (2022): 044067.
34. Y. Xu, J. Q. Gu, Y. F. Gao, Q. L. Yang, W. Y. Liu, Z. B. Yao, Q. Xu, J. G. Han, W. L. Zhang, Broadband achromatic terahertz metalens constituted by Si-SiO2-Si hybrid meta-atoms, Adv. Opt. Mater. 33 (2023): 2302821.
